# Draft genome sequence of the Tibetan medicinal herb *Rhodiola crenulata*

**DOI:** 10.1093/gigascience/gix033

**Published:** 2017-05-05

**Authors:** Yuanyuan Fu, Liangwei Li, Shijie Hao, Rui Guan, Guangyi Fan, Chengcheng Shi, Haibo Wan, Wenbin Chen, He Zhang, Guocheng Liu, Jihua Wang, Lulin Ma, Jianling You, Xuemei Ni, Zhen Yue, Xun Xu, Xiao Sun, Xin Liu, Simon Ming-Yuen Lee

**Affiliations:** 1 State Key Laboratory of Bioelectronics, School of Biological Sciences and Medical Engineering, Southeast University, Nanjing 210096, China; 2 BGI-Shenzhen, Bei Shan Industrial Zone, Yantian District, Shenzhen, Guangdong Province, 518083, P. R. China; 3 BGI-Qingdao, No. 2877, Tuanjie Road, Sino-German Ecopark, Qingdao, Shandong Province, 266555, China; 4 State Key Laboratory of Quality Research of Chinese Medicine and Institute of Chinese Medical Sciences, University of Macau, Dama Road, Macao, China; 5 Flower Research Institute of Yunnan Academy of Agricultural Sciences, National Engineering Research Center For Ornamental Horticulture, 2238 Beijing Road, Kunming, 650205, China; 6 The Ministry of Education Key Laboratory for Biodiversity Science and Ecological Engineering, Institute of Biodiversity Science, Institute of Botany, School of life Sciences, Fudan University, Songhu Road 2005, Shanghai, 200438, China

**Keywords:** *Rhodiola crenulata*, genomics, genome assembly, annotation

## Abstract

*Rhodiola crenulata*, a well-known medicinal Tibetan herb, is mainly grown in high-altitude regions of the Tibet, Yunnan, and Sichuan provinces in China. In the past few years, increasing numbers of studies have been published on the potential pharmacological activities of *R. crenulata*, strengthening our understanding into its putitive active ingredient composition, pharmacological activity, and mechanism of action. These findings also provide strong evidence supporting the important medicinal and economical value of *R. crenulata*. Consequently, some *Rhodiola* species are becoming endangered because of overexploitation and environmental destruction. However, little is known about the genetic and genomic information of any *Rhodiola* species. Here we report the first draft assembly ofthe *R. crenulata* genome, which was 344.5 Mb (25.7 Mb Ns), accounting for 82% of the estimated genome size, with a scaffold N50 length of 144.7 kb and a contig N50 length of 25.4 kb. The *R. crenulata* genome is not only highly heterozygous but also highly repetitive, with ratios of 1.12% and 66.15%, respectively, based on the *k*-mer analysis. Furthermore, 226.6 Mb of transposable elements were detected, of which 77.03% were long terminal repeats. In total, 31 517 protein-coding genes were identified, capturing 86.72% of expected plant genes in BUSCO. Additionally, 79.73% of protein-coding genes were functionally annotated. *R. crenulata* is an important medicinal plant and also a potentially interesting model species for studying the adaptability of *Rhodiola* species to extreme environments. The genomic sequences of *R. crenulata* will be useful for understanding the evolutionary mechanism of the stress resistance gene and the biosynthesis pathways of the different medicinal ingredients, for example, salidroside in *R. crenulata*.

## Data Description

### Background information

Genus *Rhodiola*, in the family *Crassulaceae*, is a perennial herbaceous flowering plant and is mainly grown in the cool climate of subarctic areas, such as North America, Northern and Central Europe, and mountainous regions of southwest and northwest China. In general, *Rhodiola* species have similar morphology, causing difficulty and confusion in their taxonomic identification and classification [1]. Although many *Rhodiola* species have been used as traditional medicines for a long time, some being widely used for therapies of cardiovascular disease, hypobaric hypoxia, microbial infection, tumour and muscular weakness, the precise pharmacological mechanisms of actions are still unclear [1–6]. In China, in comparison with other *Rhodiola* species, *R. crenulata* is the most popular and in demand, but the supply of *R. crenulata* is limited due to its stringent growing requirement. The high selling price of *R. crenulata* causes serious problems of *R. crenulata* adulteration in the market. In order to improve the understanding of *Rhodiola* species, we have sequenced the whole genome of *R. crenulata*, and have subsequently completed the genomic assembly and annotation.

### Sample collection and sequencing

**Figure 1:**
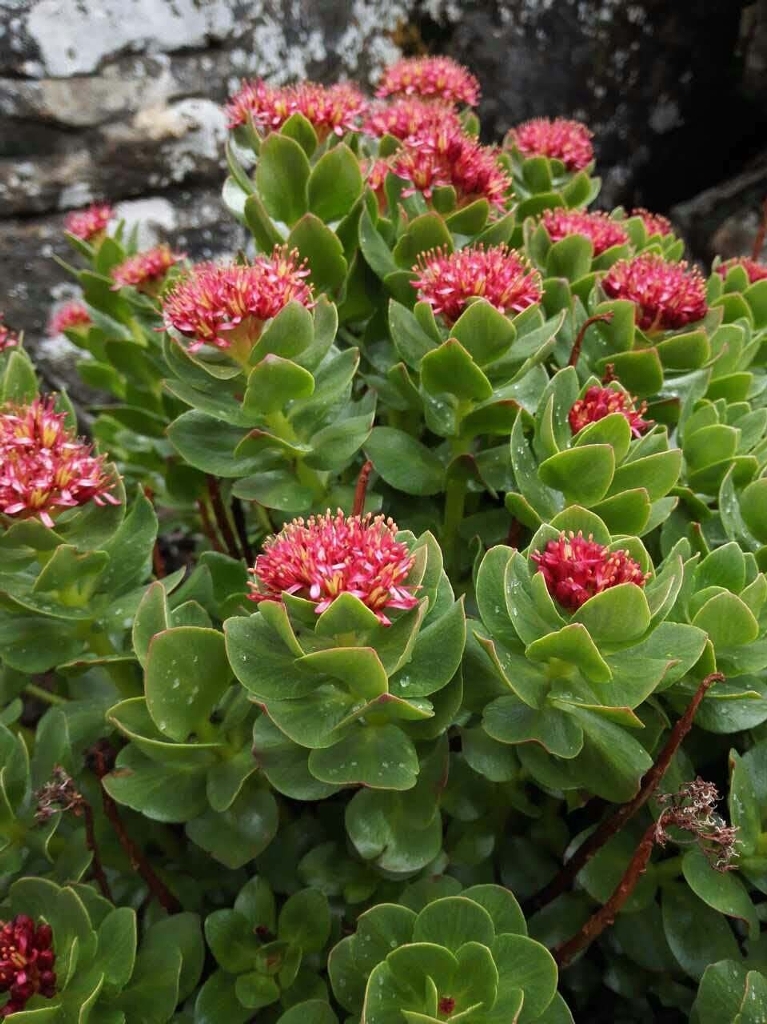
Example of *R. crenulata* (image from Shifeng Li).

According to *protocol 1* (Additional File 2), genomic DNA was isolated from the leaf tissue of a single male *R. crenulata* (NCBI taxonomy ID: 242839)(Fig. 1), which was collected from Shangri-La, located in the northwest of Yunnan province, China. Three paired-end libraries with insert sizes of 250 bp, 500 bp, and 800 bp and three mate-pair libraries (5 kb, 10 kb, and 20 kb) were subsequently constructed with the standard protocol provided by Illumina (San Diego, CA, USA) and sequenced on an Illumina HiSeq 2000/4000 platform using a whole genome shotgun sequencing (WGS) strategy. A total of 162.08 Gb (∼380X) of raw sequence reads were generated (Additional File 1: Table S1). To reduce the effect of sequencing errors to the assembly, SOAPfilter (v. 2.2), a package from SOAPdenovo2 (SOAPdenovo2, RRID:SCR_014986) [7], was used to filter reads with adapters, low quality, undersize insert size, and PCR duplication. Finally, 123.47 Gb (∼290X)of clean data were obtained (Additional File 1: Table S1).

RNA was extracted from the root, stem, and leaf tissues, respectively, of a single male *R. crenulata*, which was collected from the Jade Dragon Snow Mountain, located in the northwest of Yunnan province, China, according to the *protocol 2* (Additional File 2). Single-end libraries were constructed subsequently using standard protocol provided by BGI (BGI-Shenzhen) and then sequenced on the BGISEQ-500 platform [8,9]. In total, 13.54 Gb of raw data was obtained, and after filtering by SOAPnuke (v. 1.5.6; https://github.com/BGI-flexlab/SOAPnuke), we finally produced 13.23 Gb of high-quality clean data (Additional File 1: Table S2). In this study, different sequencing platforms were used, taking into consideration the efficiency of data generation and also allowing the consistency of data for analysis.

### Assembly

First, the genome size, 420.2 Mb, was estimated based on the 17-mer analysis [10] using 34.4 Gbof clean data from 250 bp insert library, as well as the repetitive and heterozygous ratio with 66.15% and 1.12%, respectively (Additional File 1: Table S3; Fig. S1). We also found that our estimated genome size of *R. crenulata* was relatively close to the median genome size of species inthe family *Crassulaceae* based on existing data in the C-values database [11], which range from 142 Mb to 8.9 Gb (Additional File 1: Table S4). Given the high heterozygosity, Platanus (v. 1.2.4) [12],which is efficient for the assembly of highly heterozygous genomes, was used to assemble the genome by performing “assemble, scaffold, gap_close” modes orderly with “k = 35.” As a result, 345.1 Mb (containing 65.9 Mb Ns) of draft assembly with a contig N50 length of 6.3 kb and a scaffold N50 length of 145.1 kb was generated (Additional File 1: Table S5). To further improve the quality of our assembly genome, GapCloser (v. 1.10) [7] was implemented with all six libraries of data. Finally, we obtained the 344.5 Mb (containing 25.7 Mb Ns) of assembly genome, representing 82% of the estimated genome size, with contig and scaffold N50 lengths of 25.4 kb and 144.7 kb, respectively (Table 1). Meanwhile, we also ran other prevalent *de novo* assemblers, such as SOAPdenovo2 [7]and ABySS (v. 1.9.0; ABySS, RRID:SCR_010709) [13], with various modifications of parameters. But the results based on these assemblers were not better (Additional File 1: Table S5). More methodological information is available in *protocol 3* (Additional File 2).

**Table 1:** Statistics of the final assembly using Platanus and Gapcloser.

Type	Scaffold	Contig
Total number	150 003	161 878
Total length (bp)	344 513 827	318 807 120
N50 length (bp)	144 749	25 360
N90 length (bp)	1003	877
Max length (bp)	1 309 315	300 573
GC content (%)	39.68	39.68

### Repeat annotation and gene prediction

**Figure 2:**
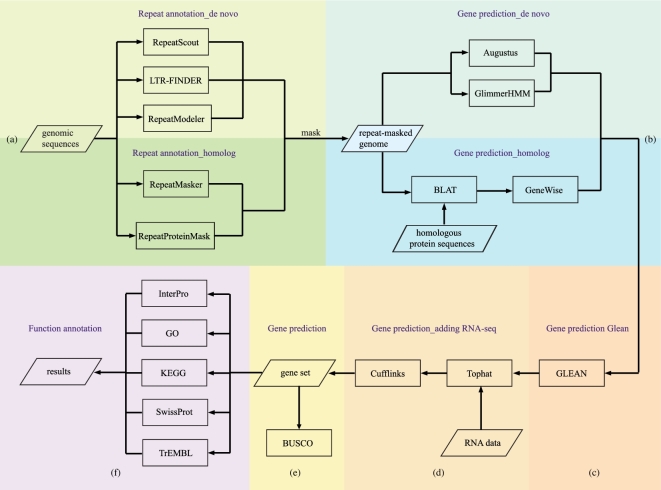
An overview of the annotation workflow. The workflow begins with assembled genomic sequences, and it produces results of the repeat annotation, protein-coding gene prediction, and functional annotation. **(a)** Repeat annotation: repeats in the genome are detected in two different methods: *de novo* and homolog based. In the *de novo* method, RepeatScout, LTR-FINDER, and RepeatModeler are used to build *de novo* repeat libraries and further classified by RepeatMasker. In the homolog-based method, RepeatMasker and RepeatProteinMask are performed to search TEs by aligning sequences against existing libraries. **(b)** Gene prediction: before the gene prediction, TEs are totally masked. Augustus and GlimmerHMM are used to perform *de novo* prediction; BLAT and GeneWise are executed to predict gene models based on homologous protein sequences. **(c)** GLEAN is performed to obtain a consensus gene set. **(d)** In combination with the clean RNA sequenced reads, a more comprehensive gene set is integrated finally. **(e)** Estimation of the completeness of the gene set using BUSCO. **(f)** Functional annotation.

*De novo* and homolog-based methods were conducted in combination to identify the transposable elements (TEs) and predict the protein-coding genes inthe *R. crenulata* genome according to *protocol 3* (Additional File 2), which is also illustrated in Fig. 2.

**Figure 3:**
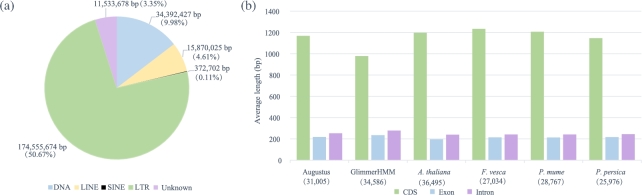
Summary statistics of the repeats and gene models. **(a)** The lengths of different types of TEs and proportions in the genome. LTR is the most predominant element. **(b)** The numbers of predicted genes and average lengths of CDS, exon, and intron predicted in different methods. The green, blue, and purple bars represent the CDS, exon, and intron, respectively. The gene numbers in each *de novo* or homolog-based method are listed in parentheses.

Briefly, in terms of the repeat detection, first, RepeatScout (v. 1.0.5; RepeatScout, RRID:SCR_014653) [14], LTR-FINDER (v. 1.0.5) [15], and RepeatModeler (v. 1.0.5) [16] were used to builda *de novo* library on the basis of our genome sequences, and then, by using the library as database, RepeatMasker (v. 3.3.0; RepeatMasker, RRID:SCR_012954) [16] was utilized to classify the types of repetitive sequences (Additional File 1: Table S6). On the other hand, TEs in DNA and protein levels were identified by aligning genome sequences against the Repbase TE library (v. 17.01) [17, 18] and TE protein database with RepeatMasker and RepeatProteinMask (v. 3.3.0) (Additional File 1: Table S7)[16]. Overall, 226.6 Mb of TEs (65.77% of the assembly) were detected, containing 174.6 Mb (50.67% of the assembly) of LTR (Fig. 3a; Additional File 1: Table S7).

Before gene prediction, TEs observed above were masked to reduce the interference. Regarding the *de novo* gene prediction, Augustus (v. 2.5.5; Augustus: Gene Prediction, RRID:SCR_008417) [19, 20] and GlimmerHMM (v. 3.0.1; GlimmerHMM, RRID:SCR_002654) [21] were conducted with the Arabidopsis training set, and 31 005 and 34 586 protein-coding genes were predicted, respectively (Fig. 3b; Additional File 1: Table S8). With respect to the homolog-based methods, because of the lack of accessible genome sequences in the family *Crassulaceae*, we downloaded the protein sequences of model organism *Arabidopsis thaliana* (https://www.ncbi.nlm.nih.gov/genome/?term=Arabidopsis+thaliana) and the relatively closely related species *Fragaria vesca* (https://www.ncbi.nlm.nih.gov/genome/3314?genome_assembly_id=34435), *Prunus mume* (https://www.ncbi.nlm.nih.gov/genome/13911?genome_assembly_id=44389), and *Prunus persica* (https://www.ncbi.nlm.nih.gov/genome/388?gen-ome_assembly_id=28754) in *rosids*, and then aligned these against the repeat-masked genome using BLAT [22]. GeneWise (v. 2.2.0) [23], whose algorithm was derived from a principled combination of hidden Markov models, was subsequently used to merge these mapping results and predict gene structures, resulting in 36 495, 27 034, 28 767, and 25 976 protein-coding genes, respectively. In addition, all average lengths of CDS, exon, and intron predicted in different methods were similar (Fig. 3b; Additional File 1: Table S8). We then performed GLEAN [24] to integratethe genes predicted above and got a non-redundant gene set containing 28 981 protein-coding genes. Also, we discarded those genes with an overlapping ratio of less than 0.8 when comparing with homolog-based evidence. A total of 27 107 genes remained. Additionally, to further improve credibility, sequenced transcriptomes data from three *R. crenulata* tissues were mapped to the consensus gene set by TopHat (v. 2.1.0; TopHat, RRID:SCR_013035) [25], and then Cufflinks (v. 2.2.1; Cufflinks, RRID:SCR_014597) [26] were executed to assemble and merge transcripts based on the mapping results. Finally, a gene set with 31 517 protein-coding genes was generated, of which 79.73% of genes could be functionally annotated with SWISS-PROT [27], TrEMBL [27], and KEGG (KEGG, RRID:SCR_012773) [28, 29] databases, and using InterProScan (v. 4.7; InterProScan, RRID:SCR_005829) (Additional File 1: Table S9)[30, 31].

### Completeness of the gene set and assembly

To evaluate the completeness of the gene set and assembly, BUSCO (BUSCO, RRID:SCR_015008) [32] was performed with “-OGS” and “-genome” modes, respectively. The results showed that 86.72% of reference genes were captured as complete single-copy BUSCOs when searching our gene set; meanwhile, regarding the assembly, 91.63% of the 956 expected plant genes were detected as complete (Table 2). Additionally, RNA sequence reads were mapped to our genome assembly by TopHat (v. 2.1.0) [25], and the average mapping ratio was almost 81.5% (Additional File 1: Table S10).

**Table 2:** Statistics of the BUSCO assessment.

	Gene Set		Assembly	
Types of BUSCOs	Number	Percentage	Number	Percentage
Complete single-copy BUSCOs	829	86.72	876	91.63
Fragmented BUSCOs	37	3.87	35	3.66
Missing BUSCOs	90	9.41	45	4.71
Total BUSCO groups searched	956	100	956	100

In summary, the *R. crenulata* genome that we have sequenced, assembled, and annotated here was the first published genome in the genus *Rhodiola* and family *Crassulaceae*. The *R. crenulata* genome should serve as an important resource for comparative genomic studies, for further investigations of the adaptability of *Rhodiola* species in an extreme environment, and for the elucidation of the biosynthesis pathways of pharmacologically active metabolites in *Rhodiola* species.

## Additional files

Additional File 1: Supplementary Tables and Figures.docx

Additional File 2: Protocols.io.xls

## Abbreviations

bp: base pair; CDS: coding sequence; Gb: giga base; kb: kilo base; Mb: mega base; SRA: Sequence Read Archive; TE: transposable elements; WGS: whole genome shotgun sequencing.

## Funding

This work was supported by the National High Technology Research and Development Program of China (NO.2014AA10A602-4) and Basic Research Program Support by the Shenzhen Municipal Government (No. JCYJ20150831201123287) and Key Research & Development Program of Jiangsu Province (BE2016002-3).

## Availability of supporting data

The DNA sequencing data have been deposited into the NCBI Sequence Read Archive (SRA) under the ID SRA538315. The RNA sequencing data are under ID SRA539059. Supporting data are also available from the *GigaScience* database (*Giga*DB) [33]. DNA/RNA extraction and assembly and annotation protocols presented here are also archived in protocols.io [34].

## Conflicts of interest

The authors declare that they have no competing interests.

## Authors’ contributions

S.M.Y.L., X.L., X.S., and X.X. designed the project. Y.F., L.L., S.H., R.G., G.F., H.W., W.C., and H.Z. analyzed the data. Y.F., S.M.Y.L., X.L., G.F., and C.S. wrote the manuscript. G.L., J.W., L.M., J.Y., X.N., and Z.Y. prepared the samples and conducted the experiments.

## Supplementary Material

GIGA-D-16-00153_Original_Submission.pdf

GIGA-D-16-00153_Revision_1.pdf

Response_to_reviewer_comments_original_submission_.pdf

Response_to_reviewer_comments_Reviewer_1.pdf

Reviewer_2_Report_(revision_1).pdf

Reviewer_3_Report_(Original_Submission).pdf

Reviewer_4_Report_(Revision_1).pdf

Review_1_Report_(Original_Submission).pdf

Review_2_Report_(Original_Submission).pdf

Additional File 1: Supplementary Tables and Figures.docx

Additional File 2: Protocols.io.xls

Additional_file_3_In-house_Perl_Script.pdf
