# Supplementary material for: Draft genome sequence of the Tibetan medicinal herb Rhodiola crenulata
Source: Gigascience. 2017 May 5;6(6):1–5. doi: 10.1093/gigascience/gix033 (PMC5530320; doi:10.1093/gigascience/gix033)
Supplement: GIGA-D-16-00153_Original_Submission.pdf [file gix033_GIGA-D-16-00153_Original_Submission.pdf]

Manuscript Click here to download Manuscript Manuscript.docx

Draft genome of the Tibetan medicinal herb, *Rhodiola crenulata*

Yuanyuan Fu[1,2,3], Liangwei Li[2,3], Shijie Hao[2], Rui Guan[2,3], Guangyi Fan[2,3,4], Chengcheng Shi[2], Haibo Wan[2,3], Wenbin Chen[2], He Zhang[2,3], Guocheng Liu[2], Jihua Wang[5], Lulin Ma[5], Jianling You[6], Xuemei Ni[2], Zhen Yue[2], Xun Xu[2], Xiao Sun[1]†, Xin Liu[2]†, Simon Ming-Yuen Lee[4]†.

[1]State Key Laboratory of Bioelectronics, School of Biological Sciences and Medical Engineering, Southeast University, Nanjing 210096, China.

[2]BGI-Shenzhen, Shenzhen 518083, China.

[3]BGI-Qingdao, Qingdao 266555, China.

[4]State Key Laboratory of Quality Research in Chinese Medicine, Institute of Chinese Medical Sciences, University of Macau, Macao, China.

[5]Flower Research Institute of Yunnan Academy of Agricultural Sciences, National Engineering Research Center For Ornamental Horticulture, Kunming, 650205, China.

[6]The Ministry of Education Key Laboratory for Biodiversity Science and Ecological Engineering, Institute of Biodiversity Science, Institute of Botany, Fudan University, Shanghai, 200438, China

†Correspondence authors: Simon Ming-Yuen Lee (simonlee@umac.mo), Xin Liu (liuxin@genomics.cn), and Xiao Sun (xsun@seu.edu.cn)

Abstract

Background

Rhodiola crenulata, one of the well-known Tibetan medicinal herb, is mainly grown in

high-altitude regions of Tibet, Yunnan and Sichuan provinces in China. In the past few years, increasing published studies on pharmacological activities of *R. crenulata*, have strengthened our understanding into its active ingredient composition, pharmacological activity and mechanism of action. The findings also provided strong evidences supporting the important medicinal and economical values of *R. crenulata*. Meanwhile, some *Rhodiola* species are becoming endangered because of overexploitation and environmental destruction. However, little is known about the genetic and genomic information of any *Rhodiola* species.

Findings

Here, we reported the first draft assembly of *R. crenulata* genome, which was 344.5 Mb (25.7Mb Ns), accounting for 82% of the estimated genome size, with the scaffold N50 length of 144.7 kb and the contig N50 length of 25.4 kb. The *R. crenulata* genome was not only highly heterozygous but also highly repetitive with ratios of 1.12% and 66.15%, respectively, based on the *k*-mer analysis. Furthermore, 226.6 Mb transposable elements were detected, of which 77.03% were long terminal repeats. In total, 31,517 protein-coding genes were identified, capturing 86.72% of expected plant genes in BUSCO. Additionally, 79.73% of protein-coding genes were functionally annotated.

Conclusions

R. crenulata is an important medicinal plant and also a potentially interesting model species for studying the adaptability of *Rhodiola* species to extreme environments. The genomic sequences of *R. crenulata* would be useful for understanding the evolutionary mechanism of stress resistance gene and biosynthesis pathways of the different

medicinal ingredients for example, salidroside, in *R. crenulata*.

Keywords:

Rhodiola crenulata, Genomics, Assembly, Annotation

Data description

Background information

Genus *Rhodiola* in the family *Crassulaceae*, a perennial herbaceous flowering plant, is mainly grown in cool climate in the subarctic areas, such as North America, Northern and Central Europe, mountainous regions of southwest and northwest China. In general, *Rhodiola* species have similar morphology, causing difficulty and confusion in their taxonomic identification and classification [1]. Although many *Rhodiola* species have been used as traditional medicines for a long time, and some of them have been widely used for therapies of cardiovascular disease, hypobaric hypoxia, microbial infection, tumour and muscular weakness, the precise pharmacological mechanisms of actions are still unclear [1-6]. In China, compared with other *Rhodiola* species, *R. crenulata* is more popular and highly demanded because of its better curative effect but the supply of *R. crenulata* is more limited due to its stringent growing requirement. The higher selling price of *R. crenulata* causes a serious problem of *R. crenulata* adulteration in the market. In order to improve the understanding of *Rhodiola* species, we have sequenced the whole genome of *R. crenulata*, and have subsequently completed the genomic assembly and annotation.

Sample collection and Sequencing

According to the ***protocol 1*** (**Additional file 2**), genomic DNA was isolated from the leaf tissue of a single male *R. crenulata* (**Fig. 1**; NCBI taxonomy ID: 242839), which was collected from Shangri-La, located in the northwest of Yunnan province, China. Subsequently, three paired-end libraries with insert size 250 bp, 500 bp, 800 bp and three mate-pair libraries (5 kb, 10 kb, 20 kb) were constructed with the standard protocol provided by Illumina (San Diego, USA) and sequenced on an Illumina HiSeq 2000/4000 platform using a whole genome shotgun sequencing (WGS) strategy. A total of 162.08 Gb (~380X) raw sequence reads were generated (**Additional file 1: Table S1**). To reduce the effect of sequencing errors to the assembly, SOAPfilter (Version 2.2), a package from SOAP*denovo*2 [7], was used to filter reads with adapters, low quality, undersize insert size and PCR duplication with parameters '-y -z -p -M 2'. Finally, 123.47 Gb (~290X) clean data were obtained (**Additional file 1: Table S1**).

RNA were extracted from the root, stem and leaf tissues, respectively, of a single male *R. crenulata*, which was collected from the Jade Dragon Snow Mountain, located at the northwest of Yunnan province, China, according to the ***protocol 2*** (**Additional file 2)**. Single-end libraries were constructed subsequently using standard protocol provided by BGI (BGI-Shenzhen) and then sequenced on the BGISEQ-500 platform. Totally, 13.54 Gb raw data was obtained, and after filtering by SOAPnuke (Version 1.5.6) (https://github.com/BGI-flexlab/SOAPnuke) with parameters "-l 10 -q 0.5 -n 0.01 -f AGTCGGAGGCCAAGCGGTCTTAGGAAGACAA -Q 2", we finally got 13.23 Gb high-quality clean data (**Additional file 1: Table S2**).

Assembly

Firstly, the genome size, 420.2 Mb, was estimated based on the 17-mer analysis [8] using 34.4 Gb clean data from 250 bp-insert library, as well as the repetitive and heterozygous ratio with 66.15% and 1.12%, respectively (**Additional file 1: Table S3; Fig. S1**). Given the high heterozygosity, Platanus (Version 1.2.4) [9] ,which is efficient for the assembly of highly heterozygous genomes, was used to assemble the genome by performing "assemble, scaffold, gap_close" modes orderly with "k=35". As a result, 345.1 Mb (containing 65.9 Mb Ns) draft assembly with the contig N50 length of 6.3 kb and the scaffold N50 length of 145.1 kb was generated (**Additional file 1: Table S4**). To further improve the quality of our assembly genome, GapCloser (Version 1.10) [7] was implemented with all of six libraries data. Finally, we got the 344.5 Mb (containing 25.7 Mb Ns) of assembly genome, representing for 82% of the estimated genome size, with the contig and scaffold N50 length of 25.4 kb and 144.7 kb, respectively (**Table 1**). Meanwhile, we also ran other prevalent *de novo* assemblers, such as SOAPdenovo2 [7], ABySS (Version 1.9.0) [10] with various modifications of parameters. But the results based on these assemblers were not better (**Additional file 1: Table S4**). More methodological information is available in the ***protocol 3*** (**Additional file 2**).

Table 1. Statistics of the final assembly using Platanus and Gapcloser.

Type	Scaffold	Contig
Total number	150,003	161,878
Total length (bp)	344,513,827	318,807,120
N50 length (bp)	144,749	25,360
N90 length (bp)	1,003	877

Max length (bp)	1,309,315	300,573
GC content (%)	39.68	39.68

Repeat annotation and gene prediction

A combination of *de novo* and homolog-based methods were conducted to identify the transposable elements (TEs) and predict the protein-coding genes in *R. crenulata* genome according to the ***protocol 3*** (**Additional file 2**), which was also illustrated in **Fig. 2**.

Briefly, in terms of the repeats detection, firstly, RepeatScout (Version 1.0.5) [11], LTR-FINDER (Version 1.0.5) [12] and RepeatModeler (Version 1.0.5) [13] were used to build *de novo* library on the basis of our genome sequences and then by using the library as database, RepeatMasker (Version 3.3.0) [13] was utilized to classify the types of repetitive sequences (**Additional file 1: Table S5**). On the other hand, TEs in DNA and protein levels were identified by aligning genome sequences against Repbase TE library (Version 17.01) [14, 15] and TE protein database with RepeatMasker and RepeatProteinMask (Version 3.3.0) [13] (**Additional file 1: Table S6**). Overall, 226.6 Mb of TEs (65.77% of the assembly) were detected, containing 174.6 Mb (50.67% of the assembly) LTR (**Fig. 3a; Additional file 1: Table S6**).

Before gene prediction, TEs observed above were masked to reduce the interference. Regarding the *de novo* gene prediction, Augustus (Version 2.5.5) [16, 17] and GlimmerHMM (Version 3.0.1) [18] were conducted with Arabidopsis training set, and 31,005 and 34,586 protein-coding genes were predicted, respectively (**Fig. 3b**; **Additional file 1: Table S7**). With respect to the homolog-based methods, because of

the lack of accessible genome sequences in family *Crassulaceae*, we downloaded the protein sequences of model organism *Arabidopsis thaliana* (https://www.ncbi.nlm.nih.gov/genome/?term=Arabidopsis+thaliana) and relatively close-related species – *Fragaria vesca* (https://www.ncbi.nlm.nih.gov/genome/3314?genome_assembly_id=34435), *Prunus mume* (https://www.ncbi.nlm.nih.gov/genome/13911?genome_assembly_id=44389) and *Prunus persica* (https://www.ncbi.nlm.nih.gov/genome/388?genome_assembly_id=28754) in *rosids*, and then aligned these against the repeat-masked genome using BLAT [19]. GeneWise (Version 2.2.0) [20], whose algorithm was derived from a principled combination of hidden Markov models, was subsequently used to merge these mapping results and predict gene structures, resulting in 36,495, 27,034, 28,767 and 25,976 protein-coding genes, respectively. In addition, each average length of CDS, exon and intron predicted in different methods were similar (**Fig. 3b**; **Additional file 1: Table S7**). Then we performed GLEAN [21] to integrate genes predicted above and got a non-redundant gene set, containing 28,981 protein-coding genes. Also, we discarded those genes with overlapping ratio less than 0.8 when comparing with homolog-based evidence. 27,107 genes were remained. Additionally, to further improve the credibility, sequenced transcriptomes data from three *R. crenulata* tissues were mapped to the consensus gene set by TopHat (Version 2.1.0) [22], and then Cufflinks (Version 2.2.1) [23] was executed to assemble and merge transcripts based on the mapping results. Finally, the gene set with 31,517 protein-coding genes was generated, of which 79.73% genes can

be functional annotation with SWISS-PROT [24], TrEMBL [24] and KEGG [25, 26] databases, and using InterProScan (Version 4.7) [27, 28] (**Additional file 1: Table S8**).

Completeness of the gene set and assembly

To evaluate the completeness of the gene set and assembly, BUSCO [29] was performed with "-OGS" and "-genome" modes, respectively. The results showed that 86.72% of reference genes were captured as complete single-copy BUSCOs when searching our gene set; meanwhile, regarding the assembly, 91.63% of the 956 expected plant genes were detected as complete (**Table 2**). Additionally, RNA sequence reads were mapped to our genome assembly by TopHat (Version 2.1.0) [22] and the average mapping ratio was almost 81.5% (**Additional file 1: Table S9**).

Table 2. Statistics of the BUSCO assessment.

	**Gene set**		**Assembly**	
**Types of BUSCOs**	**Number**	**Percentage (%)**	**Number**	**Percentage (%)**
Complete Single-copy BUSCOs	829	86.72	876	91.63
Fragmented BUSCOs	37	3.87	35	3.66
Missing BUSCOs	90	9.41	45	4.71
Total BUSCO groups searched	956	100	956	100

In summary, the *R. crenulata* genome that we have sequenced, assembled and annotated here, was the first one in the Genus *Rhodiola*, and even in the family *Crassulaceae*. The *R. crenulata* genome would serve as an important resource for

comparative genomic study and also further investigation of the adaptability of *Rhodiola* species in extreme environment and the biosynthesis pathways of pharmacologically active metabolites in *Rhodiola* species.

Figure legends

Figure 1. Example of *R. crenulata* (image from Shifeng Li).

Figure 2. An overview of the annotation workflow. The workflow begins with assembled genomic sequences, and it produces results of the repeat annotation, protein-coding gene prediction and functional annotation. (a) Repeat annotation. Repeats in the genome are detected in two different methods: *de novo* and homolog-based. In the *de novo* methods, RepeatScout, LTR-FINDER and RepeatModeler are used to build *de novo* repeat libraries and further classified by RepeatMasker; In the homolog-based methods, RepeatMasker and RepeatProteinMask are performed to search TEs by aligning sequences against existed libraries. (b) Gene prediction. Before the gene prediction, TEs are totally masked. Augustus and GlimmerHMM are used to perform *de novo* prediction; BLAT and GeneWise are executed to predict gene models based on the homologous protein sequences. (c) GLEAN is performed to obtain consensus gene set. (d) In combination with the clean RNA sequenced reads, a more comprehensive gene set is integrated finally. (e) Estimation of the completeness of gene set by using BUSCO. (f) Functional annotation.

Figure3. Summary statistics of the repeats and gene models. (a) The lengths of different types of TEs and proportions in genome. LTR is the most predominant

elements. (b) The numbers of predicted genes and average lengths of CDS, exon and intron predicted in different methods. The green, blue and purple bars represent the CDS, exon and intron, respectively. The gene numbers in each *de novo* or homolog-based method are listed in parentheses.

Availability of supporting data

The DNA sequencing data have been deposited into NCBI Sequence Read Archive (SRA) under ID SRA538315. The RNA sequencing data are under ID SRA539059. Supporting data are available at *Giga*DB: ftp://gigadb_private2@climb.genomics.cn.

Abbreviations

bp: base pair, kb: kilo base, Mb: mega base, Gb: giga base, CDS: coding sequence

Additional files

Additional file 1: Supplementary Tables and Figures.docx

Additional file 2: Protocols.io.xls

Acknowledgements

This work was supported by the National High Technology Research and Development Program of China (NO.2014AA10A602-4) and Basic Research Program Support by Shenzhen Municipal Government (NO. JCYJ20150831201123287).

Competing interests

The authors declare that they have no competing interests.

Authors' contributions

S. M.Y.L, X.L, X.S and X.X designed the project. Y.F, L.L, S.H, R.G, G.F, H.W, W.C,

H.Z analyzed the data. Y.F, S.M.Y.L, X.L, G.F, C.S wrote the manuscript. G.L, J.W, L.M, J.Y, X.N, Z.Y prepared the samples and conducted the experiments.

References

1. Recio MC, Giner RM, Manez S. Immunmodulatory and Antiproliferative Properties of Rhodiola Species. Planta medica. 2016;82(11-12):952-60. doi:10.1055/s-0042-107254.

2. Zhu C, Guan F, Wang C, Jin LH. The protective effects of Rhodiola crenulata extracts on Drosophila melanogaster gut immunity induced by bacteria and SDS toxicity. Phytotherapy research : PTR. 2014;28(12):1861-6. doi:10.1002/ptr.5215.

3. Bassa LM, Jacobs C, Gregory K, Henchey E, Ser-Dolansky J, Schneider SS. Rhodiola crenulata induces an early estrogenic response and reduces proliferation and tumorsphere formation over time in MCF7 breast cancer cells. Phytomedicine : international journal of phytotherapy and phytopharmacology. 2016;23(1):87-94. doi:10.1016/j.phymed.2015.11.014.

4. Dudek MC, Wong KE, Bassa LM, Mora MC, Ser-Dolansky J, Henneberry JM et al. Antineoplastic effects of Rhodiola crenulata treatment on B16-F10 melanoma. Tumour biology : the journal of the International Society for Oncodevelopmental Biology and Medicine. 2015;36(12):9795-805. doi:10.1007/s13277-015-3742-2.

5. Cai Z, Li W, Wang H, Yan W, Zhou Y, Wang G et al. Antitumor effects of a purified polysaccharide from Rhodiola rosea and its action mechanism. Carbohydrate polymers. 2012;90(1):296-300. doi:10.1016/j.carbpol.2012.05.039.

6. Panossian A, Wikman G, Sarris J. Rosenroot (Rhodiola rosea): traditional use, chemical composition, pharmacology and clinical efficacy. Phytomedicine : international journal of phytotherapy and phytopharmacology. 2010;17(7):481-93. doi:10.1016/j.phymed.2010.02.002.

7. Luo R, Liu B, Xie Y, Li Z, Huang W, Yuan J et al. SOAPdenovo2: an empirically improved memory-efficient short-read de novo assembler. GigaScience. 2012;1(1):18. doi:10.1186/2047-217X-1-18.

8. Li R, Fan W, Tian G, Zhu H, He L, Cai J et al. The sequence and de novo assembly of the giant panda genome. Nature. 2010;463(7279):311-7. doi:10.1038/nature08696.

9. Kajitani R, Toshimoto K, Noguchi H, Toyoda A, Ogura Y, Okuno M et al. Efficient

de novo assembly of highly heterozygous genomes from whole-genome shotgun short reads. Genome research. 2014;24(8):1384-95. doi:10.1101/gr.170720.113.
10. Simpson JT, Wong K, Jackman SD, Schein JE, Jones SJ, Birol I. ABySS: a parallel assembler for short read sequence data. Genome research. 2009;19(6):1117-23. doi:10.1101/gr.089532.108.
11. Price AL, Jones NC, Pevzner PA. De novo identification of repeat families in large genomes. Bioinformatics. 2005;21 Suppl 1:i351-8. doi:10.1093/bioinformatics/bti1018.
12. Xu Z, Wang H. LTR_FINDER: an efficient tool for the prediction of full-length LTR retrotransposons. Nucleic acids research. 2007;35(Web Server issue):W265-8. doi:10.1093/nar/gkm286.
13. Tarailo-Graovac M, Chen N. Using RepeatMasker to identify repetitive elements in genomic sequences. Current protocols in bioinformatics. 2009;Chapter 4:Unit 4 10. doi:10.1002/0471250953.bi0410s25.
14. Bao W, Kojima KK, Kohany O. Repbase Update, a database of repetitive elements in eukaryotic genomes. Mobile DNA. 2015;6:11. doi:10.1186/s13100-015-0041-9.
15. Jurka J, Kapitonov VV, Pavlicek A, Klonowski P, Kohany O, Walichiewicz J. Repbase Update, a database of eukaryotic repetitive elements. Cytogenetic and genome research. 2005;110(1-4):462-7. doi:10.1159/000084979.
16. Stanke M, Keller O, Gunduz I, Hayes A, Waack S, Morgenstern B. AUGUSTUS: ab initio prediction of alternative transcripts. Nucleic acids research. 2006;34(Web Server issue):W435-9. doi:10.1093/nar/gkl200.
17. Keller O, Kollmar M, Stanke M, Waack S. A novel hybrid gene prediction method employing protein multiple sequence alignments. Bioinformatics. 2011;27(6):757-63. doi:10.1093/bioinformatics/btr010.
18. Majoros WH, Pertea M, Salzberg SL. TigrScan and GlimmerHMM: two open source ab initio eukaryotic gene-finders. Bioinformatics. 2004;20(16):2878-9. doi:10.1093/bioinformatics/bth315.
19. Kent WJ. BLAT--the BLAST-like alignment tool. Genome research. 2002;12(4):656-64. doi:10.1101/gr.229202. Article published online before March 2002.
20. Birney E, Clamp M, Durbin R. GeneWise and Genomewise. Genome research. 2004;14(5):988-95. doi:10.1101/gr.1865504.
21. Elsik CG, Mackey AJ, Reese JT, Milshina NV, Roos DS, Weinstock GM. Creating

a honey bee consensus gene set. Genome biology. 2007;8(1):R13. doi:10.1186/gb-2007-8-1-r13.

22. Trapnell C, Pachter L, Salzberg SL. TopHat: discovering splice junctions with RNA-Seq. Bioinformatics. 2009;25(9):1105-11. doi:10.1093/bioinformatics/btp120.

23. Trapnell C, Roberts A, Goff L, Pertea G, Kim D, Kelley DR et al. Differential gene and transcript expression analysis of RNA-seq experiments with TopHat and Cufflinks. Nature protocols. 2012;7(3):562-78. doi:10.1038/nprot.2012.016.

24. Bairoch A, Apweiler R. The SWISS-PROT protein sequence database and its supplement TrEMBL in 2000. Nucleic acids research. 2000;28(1):45-8.

25. Kanehisa M, Goto S, Sato Y, Kawashima M, Furumichi M, Tanabe M. Data, information, knowledge and principle: back to metabolism in KEGG. Nucleic acids research. 2014;42(Database issue):D199-205. doi:10.1093/nar/gkt1076.

26. Kanehisa M, Goto S. KEGG: kyoto encyclopedia of genes and genomes. Nucleic acids research. 2000;28(1):27-30.

27. Jones P, Binns D, Chang HY, Fraser M, Li W, McAnulla C et al. InterProScan 5: genome-scale protein function classification. Bioinformatics. 2014;30(9):1236-40. doi:10.1093/bioinformatics/btu031.

28. Zdobnov EM, Apweiler R. InterProScan--an integration platform for the signature-recognition methods in InterPro. Bioinformatics. 2001;17(9):847-8.

29. Simao FA, Waterhouse RM, Ioannidis P, Kriventseva EV, Zdobnov EM. BUSCO: assessing genome assembly and annotation completeness with single-copy orthologs. Bioinformatics. 2015;31(19):3210-2. doi:10.1093/bioinformatics/btv351.

Figure

Click here to download Figure Figure 1.jpg

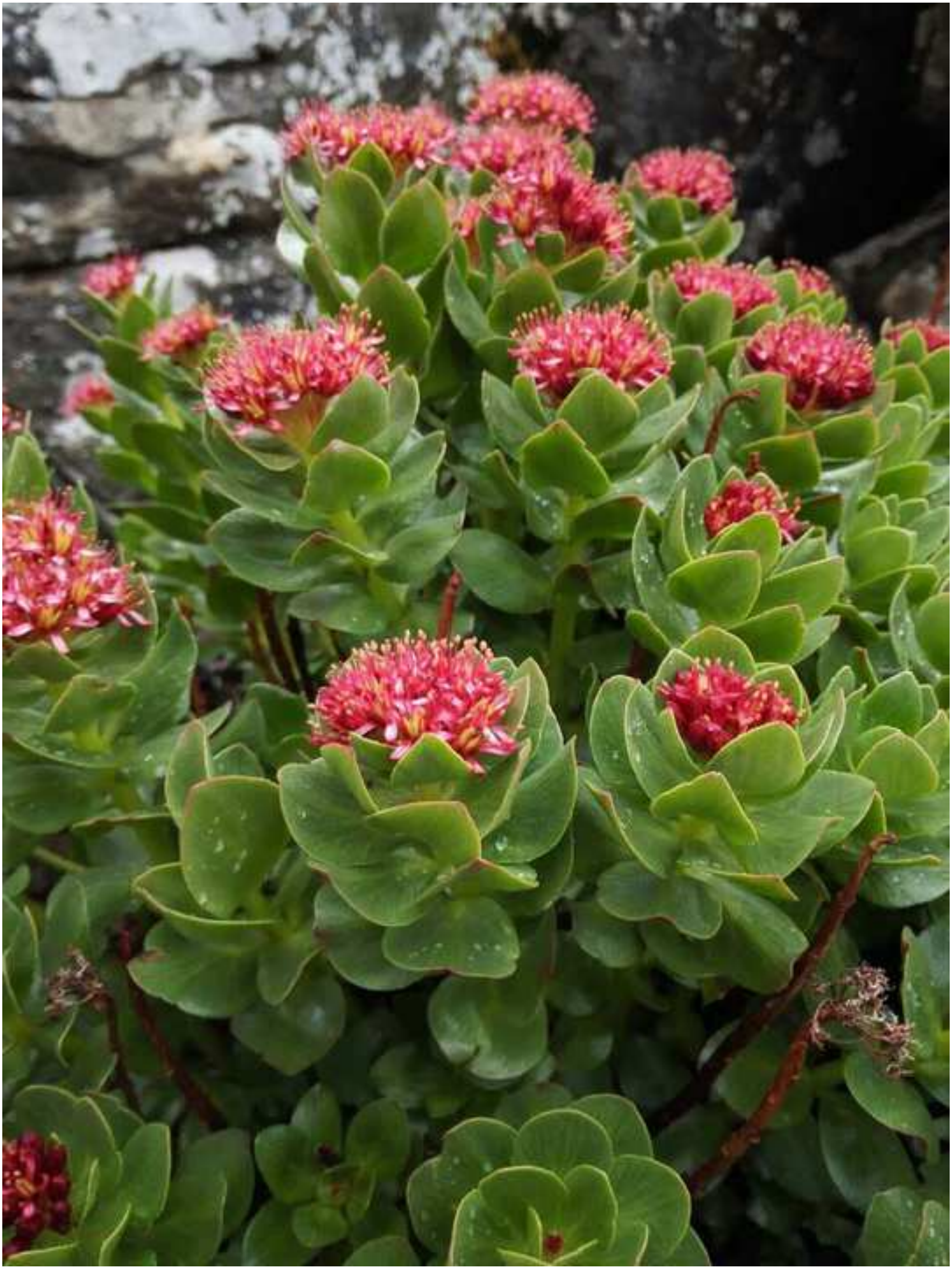

Figure

Click here to download Figure Figure 2.pdf

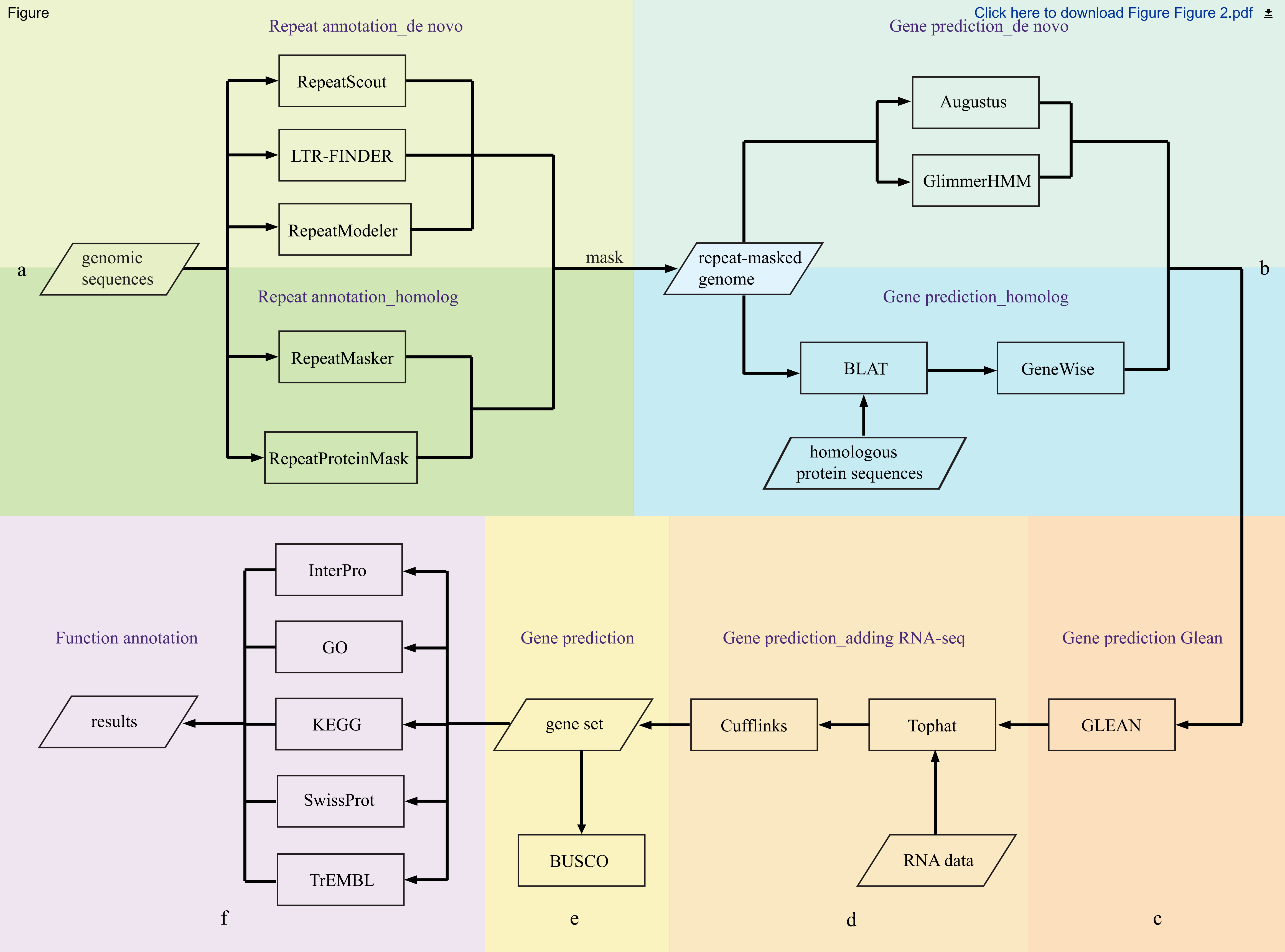

Figure

Click here to download Figure Figure 3.pdf

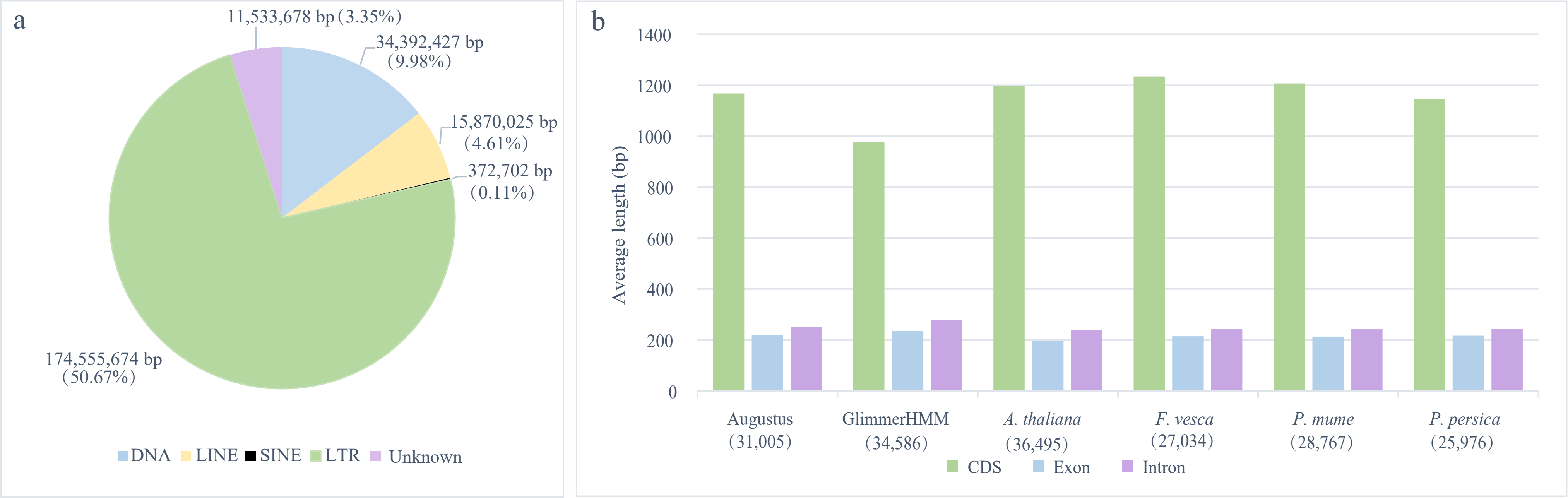

Click here to access/download
Supplementary Material
Additional file1_Supplementary Tables and Figures.docx

Click here to access/download
Supplementary Material
Additional file 2_Protocols.io.xlsx

Personal Cover Click here to download Personal Cover CoverLetter.docx

Dear Editor,

Please find enclosed our manuscript entitled "***Draft genome of the Tibetan medicinal herb, Rhodiola crenulata***", which we wish to submit for publication as a DataNote in *GigaScience*. All co-authors have approved the final version of this manuscript and there is no financial interest or other conflict to declare. We certify that the submission is original work and is not under review with another journal.

Rhodiola crenulata, one of the well-known Tibetan medicinal herb, is mainly grown in high-altitude regions of Tibet, Yunnan and Sichuan provinces in China. In the past few years, increasing published studies on pharmacological activities of *R. crenulata*, have strengthened our understanding into its active ingredient composition, pharmacological activity and mechanism of action. The findings also provided strong evidences supporting the important medicinal and economical values of *R. crenulata*. Meanwhile, some *Rhodiola* species are becoming endangered because of overexploitation and environmental destruction. However, little is known about the genetic and genomic information of any *Rhodiola* species. Here, we sequenced and assembled the genome sequences of *R. crenulata*, which is also the first sequenced species in family *Crassulaceae*. A total of 162.08 Gb (~380X) raw sequence reads were generated and 344.5 Mb (containing 25.7 Mb Ns) of assembly genome, representing for 82% of the estimated genome size, with the contig and scaffold N50 length of 25.4 kb and 144.7 kb, respectively, was obtained. We also provided a detailed assessment of the genome completeness, and carried out transposable element, protein-coding genes prediction for the genome assembly. The predicted genes were also functionally annotated.

We believe that the *R. crenulata* genome that we have sequenced, assembled and annotated here, would serve as an important resource for comparative genomic study and also further investigation of the adaptability of *Rhodiola* species in extreme

environment and the biosynthesis pathways of pharmacologically active metabolites in *Rhodiola* species.

I hope you will find our study of interest and look forward to hearing from you.

Sincerely yours,

Xin Liu, PhD
liuxin@genomics.cn
BGI-Shenzhen, Shenzhen, 518083, China
