# Supplementary material for: Draft genome sequence of the Tibetan medicinal herb Rhodiola crenulata
Source: Gigascience. 2017 May 5;6(6):1–5. doi: 10.1093/gigascience/gix033 (PMC5530320; doi:10.1093/gigascience/gix033)
Supplement: GIGA-D-16-00153_Revision_1.pdf [file gix033_GIGA-D-16-00153_Revision_1.pdf]

Manuscript

Click here to download Manuscript Manuscript_revised(1).DOCX

Draft genome sequence of the Tibetan medicinal herb, *Rhodiola crenulata*

Yuanyuan Fu[1,2,3], Liangwei Li[2,3], Shijie Hao[2], Rui Guan[2,3], Guangyi Fan[2,3,4], Chengcheng Shi[2], Haibo Wan[2,3], Wenbin Chen[2], He Zhang[2,3], Guocheng Liu[2], Jihua Wang[5], Lulin Ma[5], Jianling You[6], Xuemei Ni[2], Zhen Yue[2], Xun Xu[2], Xiao Sun[1]†, Xin Liu[2]†, Simon Ming-Yuen Lee[4]†.

[1]State Key Laboratory of Bioelectronics, School of Biological Sciences and Medical Engineering, Southeast University, Nanjing 210096, China.

[2]BGI-Shenzhen, Shenzhen 518083, China.

[3]BGI-Qingdao, Qingdao 266555, China.

[4]State Key Laboratory of Quality Research in Chinese Medicine, Institute of Chinese Medical Sciences, University of Macau, Macao, China.

[5]Flower Research Institute of Yunnan Academy of Agricultural Sciences, National Engineering Research Center For Ornamental Horticulture, Kunming, 650205, China.

[6]The Ministry of Education Key Laboratory for Biodiversity Science and Ecological Engineering, Institute of Biodiversity Science, Institute of Botany, Fudan University, Shanghai, 200438, China

†Correspondence authors: Simon Ming-Yuen Lee (simonlee@umac.mo), Xin Liu (liuxin@genomics.cn), and Xiao Sun (xsun@seu.edu.cn)

Abstract

Background

Rhodiola crenulata, a well-known medicinal Tibetan herb, is mainly grown in high-altitude regions of Tibet, Yunnan and Sichuan provinces in China. In the past few years increasing numbers of studies have been published on the potential pharmacological activities of *R. crenulata*, strengthening our understanding into its putitive active ingredient composition, pharmacological activity and mechanism of action. These findings also provide strong evidence supporting the important medicinal and economical value of *R. crenulata*. Consequently, some *Rhodiola* species are becoming endangered because of overexploitation and environmental destruction. However, little is known about the genetic and genomic information of any *Rhodiola* species.

Findings

Here we report the first draft assembly of *R. crenulata* genome, which was 344.5 Mb (25.7Mb Ns), accounting for 82% of the estimated genome size, with a scaffold N50 length of 144.7 kb and the contig N50 length of 25.4 kb. The *R. crenulata* genome is not only highly heterozygous but also highly repetitive with ratios of 1.12% and 66.15%, respectively, based on the *k*-mer analysis. Furthermore, 226.6 Mb of transposable elements were detected, of which 77.03% were long terminal repeats. In total, 31,517 protein-coding genes were identified, capturing 86.72% of expected plant genes in BUSCO. Additionally, 79.73% of protein-coding genes were functionally annotated.

Conclusions

R. crenulata is an important medicinal plant and also a potentially interesting model

species for studying the adaptability of *Rhodiola* species to extreme environments. The genomic sequences of *R. crenulata* will be useful for understanding the evolutionary mechanism of stress resistance gene and biosynthesis pathways of the different medicinal ingredients for example, salidroside, in *R. crenulata*.

Keywords:

Rhodiola crenulata, Genomics, Genome Assembly, Annotation

Data description

Background information

Genus *Rhodiola* in the family *Crassulaceae*, is a perennial herbaceous flowering plant, and is mainly grown in the cool climate of subarctic areas, such as North America, Northern and Central Europe, mountainous regions of southwest and northwest China. In general, *Rhodiola* species have similar morphology, causing difficulty and confusion in their taxonomic identification and classification [1]. Although many *Rhodiola* species have been used as traditional medicines for a long time, and some being widely used for therapies of cardiovascular disease, hypobaric hypoxia, microbial infection, tumour and muscular weakness, the precise pharmacological mechanisms of actions are still unclear [1-6]. In China, in comparison with other *Rhodiola* species, *R. crenulata* is the most popular and in demand, but the supply of *R. crenulata* is limited due to its stringent growing requirement. The high selling price of *R. crenulata* causes serious problems of *R. crenulata* adulteration in the market. In

order to improve the understanding of *Rhodiola* species, we have sequenced the whole genome of *R. crenulata*, and have subsequently completed the genomic assembly and annotation.

Sample collection and sequencing

According to the ***protocol 1*** (**Additional file 2**), genomic DNA was isolated from the leaf tissue of a single male *R. crenulata* (**Fig. 1**; NCBI taxonomy ID: 242839), which was collected from Shangri-La, located in the northwest of Yunnan province, China. Three paired-end libraries with insert size 250 bp, 500 bp, 800 bp and three mate-pair libraries (5 kb, 10 kb, 20 kb) were subsequently constructed with the standard protocol provided by Illumina (San Diego, USA) and sequenced on an Illumina HiSeq 2000/4000 platform using a whole genome shotgun sequencing (WGS) strategy. A total of 162.08 Gb (~380X) raw sequence reads were generated (**Additional file 1: Table S1**). To reduce the effect of sequencing errors to the assembly, SOAPfilter (Version 2.2), a package from SOAP*denovo*2 (SOAPdenovo2 , RRID:SCR_014986)[7], was used to filter reads with adapters, low quality, undersize insert size and PCR duplication. Finally, 123.47 Gb (~290X) clean data were obtained (**Additional file 1: Table S1**).

RNA was extracted from the root, stem and leaf tissues, respectively, of a single male *R. crenulata*, which was collected from the Jade Dragon Snow Mountain, located in the northwest of Yunnan province, China, according to the ***protocol 2*** (**Additional file 2)**. Single-end libraries were constructed subsequently using standard protocol provided by BGI (BGI-Shenzhen) and then sequenced on the BGISEQ-500

platform[8, 9]. Totally, 13.54 Gb raw data was obtained, and after filtering by SOAPnuke (Version 1.5.6) (https://github.com/BGI-flexlab/SOAPnuke), we finally produced 13.23 Gb of high-quality clean data (**Additional file 1: Table S2**). In this study, different sequencing platforms were used taking into considering the efficiency of data generation, and also allowing the consistency of data for analysis.

Assembly

Firstly, the genome size, 420.2 Mb, was estimated based on the 17-mer analysis [10] using 34.4 Gb clean data from 250 bp-insert library, as well as the repetitive and heterozygous ratio with 66.15% and 1.12%, respectively (**Additional file 1: Table S3; Fig. S1**). We also found that our estimated genome size of *R. crenulata* was relatively close to the median genome size of species in family *Crassulaceae* based on existing data in the C-values database[11], which ranging from 142 Mb to 8.9 Gb (**Additional file 1: Table S4).** Given the high heterozygosity, Platanus (Version 1.2.4) [12] ,which is efficient for the assembly of highly heterozygous genomes, was used to assemble the genome by performing “assemble, scaffold, gap_close” modes orderly with “k=35”. As a result, 345.1 Mb (containing 65.9 Mb Ns) draft assembly with the contig N50 length of 6.3 kb and the scaffold N50 length of 145.1 kb was generated (**Additional file 1: Table S5**). To further improve the quality of our assembly genome, GapCloser (Version 1.10) [7] was implemented with all of six libraries data. Finally, we obtained the 344.5 Mb (containing 25.7 Mb Ns) of assembly genome, representing for 82% of the estimated genome size, with the contig and scaffold N50 length of 25.4 kb and 144.7 kb, respectively (**Table 1**). Meanwhile, we also ran other

prevalent *de novo* assemblers, such as SOAPdenovo2 [7], ABySS (Version 1.9.0)(ABySS , RRID:SCR_010709) [13] with various modifications of parameters. But the results based on these assemblers were not better (**Additional file 1: Table S5**). More methodological information is available in ***protocol 3*** (**Additional file 2**).

Table 1. Statistics of the final assembly using Platanus and Gapcloser.

Type	Scaffold	Contig
Total number	150,003	161,878
Total length (bp)	344,513,827	318,807,120
N50 length (bp)	144,749	25,360
N90 length (bp)	1,003	877
Max length (bp)	1,309,315	300,573
GC content (%)	39.68	39.68

Repeat annotation and gene prediction

A combination of *de novo* and homolog-based methods were conducted to identify the transposable elements (TEs) and predict the protein-coding genes in *R. crenulata* genome according to the ***protocol 3*** (**Additional file 2**), which was also illustrated in **Fig. 2**.

Briefly, in terms of the repeats detection, firstly, RepeatScout (Version 1.0.5)(RepeatScout , RRID:SCR_014653) [14], LTR-FINDER (Version 1.0.5) [15] and RepeatModeler (Version 1.0.5) [16] were used to build *de novo* library on the basis of our genome sequences and then by using the library as database, RepeatMasker (Version 3.3.0)(RepeatMasker , RRID:SCR_012954) [16] was utilized to classify the types of repetitive sequences (**Additional file 1: Table S6**). On the

other hand, TEs in DNA and protein levels were identified by aligning genome sequences against Repbase TE library (Version 17.01) [17, 18] and TE protein database with RepeatMasker and RepeatProteinMask (Version 3.3.0) [16] (**Additional file 1: Table S7**). Overall, 226.6 Mb of TEs (65.77% of the assembly) were detected, containing 174.6 Mb (50.67% of the assembly) LTR (**Fig. 3a; Additional file 1: Table S7**).

Before gene prediction, TEs observed above were masked to reduce the interference. Regarding the *de novo* gene prediction, Augustus (Version 2.5.5)(Augustus: Gene Prediction , RRID:SCR_008417) [19, 20] and GlimmerHMM (Version 3.0.1)(GlimmerHMM , RRID:SCR_002654) [21] were conducted with Arabidopsis training set, and 31,005 and 34,586 protein-coding genes were predicted, respectively (**Fig. 3b**; **Additional file 1: Table S8**). With respect to the homolog-based methods, because of the lack of accessible genome sequences in family *Crassulaceae*, we downloaded the protein sequences of model organism *Arabidopsis thaliana* (https://www.ncbi.nlm.nih.gov/genome/?term=Arabidopsis+thaliana) and relatively close-related species – *Fragaria vesca* (https://www.ncbi.nlm.nih.gov/genome/3314?genome_assembly_id=34435), *Prunus mume* (https://www.ncbi.nlm.nih.gov/genome/13911?genome_assembly_id=44389) and *Prunus persica* (https://www.ncbi.nlm.nih.gov/genome/388?genome_assembly_id=28754) in *rosids*, and then aligned these against the repeat-masked genome using BLAT [22]. GeneWise (Version 2.2.0) [23], whose algorithm was derived from a principled

combination of hidden Markov models, was subsequently used to merge these mapping results and predict gene structures, resulting in 36,495, 27,034, 28,767 and 25,976 protein-coding genes, respectively. In addition, each average length of CDS, exon and intron predicted in different methods were similar (**Fig. 3b**; **Additional file 1: Table S8**). We then performed GLEAN [24] to integrate genes predicted above and got a non-redundant gene set, containing 28,981 protein-coding genes. Also, we discarded those genes with overlapping ratio less than 0.8 when comparing with homolog-based evidence. 27,107 genes were remained. Additionally, to further improve the credibility, sequenced transcriptomes data from three *R. crenulata* tissues were mapped to the consensus gene set by TopHat (Version 2.1.0)(TopHat , RRID:SCR_013035) [25], and then Cufflinks (Version 2.2.1)(Cufflinks , RRID:SCR_014597) [26] were executed to assemble and merge transcripts based on the mapping results. Finally, a gene set with 31,517 protein-coding genes was generated, of which 79.73% genes can be functional annotation with SWISS-PROT [27], TrEMBL [27] and KEGG (KEGG , RRID:SCR_012773) [28, 29] databases, and using InterProScan (Version 4.7)(InterProScan , RRID:SCR_005829) [30, 31] (**Additional file 1: Table S9**).

Completeness of the gene set and assembly

To evaluate the completeness of the gene set and assembly, BUSCO (BUSCO , RRID:SCR_015008)[32] was performed with "-OGS" and "-genome" modes, respectively. The results showed that 86.72% of reference genes were captured as complete single-copy BUSCOs when searching our gene set; meanwhile, regarding

the assembly, 91.63% of the 956 expected plant genes were detected as complete (**Table 2**). Additionally, RNA sequence reads were mapped to our genome assembly by TopHat (Version 2.1.0) [25] and the average mapping ratio was almost 81.5% (**Additional file 1: Table S10**).

Table 2. Statistics of the BUSCO assessment.

Types of BUSCOs	Gene set		Assembly	
	**Number**	**Percentage (%)**	**Number**	**Percentage (%)**
Complete Single-copy BUSCOs	829	86.72	876	91.63
Fragmented BUSCOs	37	3.87	35	3.66
Missing BUSCOs	90	9.41	45	4.71
Total BUSCO groups searched	956	100	956	100

In summary, the *R. crenulata* genome that we have sequenced, assembled and annotated here, was the first published genome in the Genus *Rhodiola* and family *Crassulaceae*. The *R. crenulata* genome should serve as an important resource for comparative genomic studies, for further investigations of the adaptability of *Rhodiola* species in extreme environment, and to elucidate the biosynthesis pathways of pharmacologically active metabolites in *Rhodiola* species.

Figure legends

Figure 1. Example of *R. crenulata* (image from Shifeng Li).

Figure 2. An overview of the annotation workflow. The workflow begins with assembled genomic sequences, and it produces results of the repeat annotation, protein-coding gene prediction and functional annotation. (a) Repeat annotation. Repeats in the genome are detected in two different methods: *de novo* and homolog-based. In the *de novo* methods, RepeatScout, LTR-FINDER and RepeatModeler are used to build *de novo* repeat libraries and further classified by RepeatMasker; In the homolog-based methods, RepeatMasker and RepeatProteinMask are performed to search TEs by aligning sequences against existing libraries. (b) Gene prediction. Before the gene prediction, TEs are totally masked. Augustus and GlimmerHMM are used to perform *de novo* prediction; BLAT and GeneWise are executed to predict gene models based on the homologous protein sequences. (c) GLEAN is performed to obtain consensus gene set. (d) In combination with the clean RNA sequenced reads, a more comprehensive gene set is integrated finally. (e) Estimation of the completeness of gene set by using BUSCO. (f) Functional annotation.

Figure3. Summary statistics of the repeats and gene models. (a) The lengths of different types of TEs and proportions in genome. LTR is the most predominant elements. (b) The numbers of predicted genes and average lengths of CDS, exon and intron predicted in different methods. The green, blue and purple bars represent the CDS, exon and intron, respectively. The gene numbers in each *de novo* or homolog-based method are listed in parentheses.

Availability of supporting data

The DNA sequencing data have been deposited into NCBI Sequence Read Archive (SRA) under the ID SRA538315. The RNA sequencing data are under ID SRA539059. Supporting data is also available from the *GigaScience Giga*DB database[33].

Abbreviations

bp: base pair, CDS: coding sequence, Gb: giga base, kb: kilo base, Mb: mega base, SRA: Sequence Read Archive, TE: transposable elements, WGS: whole genome shotgun sequencing

Additional files

Additional file 1: Supplementary Tables and Figures.docx

Additional file 2: Protocols.io.xls

Acknowledgements

This work was supported by the National High Technology Research and Development Program of China (NO.2014AA10A602-4) and Basic Research Program Support by Shenzhen Municipal Government (NO. JCYJ20150831201123287).

Competing interests

The authors declare that they have no competing interests.

Authors' contributions

S. M.Y.L, X.L, X.S and X.X designed the project. Y.F, L.L, S.H, R.G, G.F, H.W, W.C, H.Z analyzed the data. Y.F, S.M.Y.L, X.L, G.F, C.S wrote the manuscript. G.L, J.W,

L.M, J.Y, X.N, Z.Y prepared the samples and conducted the experiments.

References

1. Recio MC, Giner RM, Manez S. Immunmodulatory and Antiproliferative Properties of Rhodiola Species. Planta medica. 2016;82(11-12):952-60. doi:10.1055/s-0042-107254.

2. Zhu C, Guan F, Wang C, Jin LH. The protective effects of Rhodiola crenulata extracts on Drosophila melanogaster gut immunity induced by bacteria and SDS toxicity. Phytotherapy research : PTR. 2014;28(12):1861-6. doi:10.1002/ptr.5215.

3. Bassa LM, Jacobs C, Gregory K, Henchey E, Ser-Dolansky J, Schneider SS. Rhodiola crenulata induces an early estrogenic response and reduces proliferation and tumorsphere formation over time in MCF7 breast cancer cells. Phytomedicine : international journal of phytotherapy and phytopharmacology. 2016;23(1):87-94. doi:10.1016/j.phymed.2015.11.014.

4. Dudek MC, Wong KE, Bassa LM, Mora MC, Ser-Dolansky J, Henneberry JM et al. Antineoplastic effects of Rhodiola crenulata treatment on B16-F10 melanoma. Tumour biology : the journal of the International Society for Oncodevelopmental Biology and Medicine. 2015;36(12):9795-805. doi:10.1007/s13277-015-3742-2.

5. Cai Z, Li W, Wang H, Yan W, Zhou Y, Wang G et al. Antitumor effects of a purified polysaccharide from Rhodiola rosea and its action mechanism. Carbohydrate polymers. 2012;90(1):296-300. doi:10.1016/j.carbpol.2012.05.039.

6. Panossian A, Wikman G, Sarris J. Rosenroot (Rhodiola rosea): traditional use, chemical composition, pharmacology and clinical efficacy. Phytomedicine : international journal of phytotherapy and phytopharmacology. 2010;17(7):481-93. doi:10.1016/j.phymed.2010.02.002.

7. Luo R, Liu B, Xie Y, Li Z, Huang W, Yuan J et al. SOAPdenovo2: an empirically improved memory-efficient short-read de novo assembler. GigaScience. 2012;1(1):18. doi:10.1186/2047-217X-1-18.

8. Goodwin S, McPherson JD, McCombie WR. Coming of age: ten years of next-generation sequencing technologies. Nature reviews Genetics.

2016;17(6):333-51. doi:10.1038/nrg.2016.49.
9. Yuzuki D. BGISEQ-500 debuts at the International Congress of Genomics 10. Next Generation Technologist. 2015.
10. Li R, Fan W, Tian G, Zhu H, He L, Cai J et al. The sequence and de novo assembly of the giant panda genome. Nature. 2010;463(7279):311-7. doi:10.1038/nature08696.
11. Bennett M, Leitch I. Plant DNA C-values database (release 6.0, Dec. 2012). WWW document] URL http://data kew org/cvalues/[accessed 14 October 2014]. 2012.
12. Kajitani R, Toshimoto K, Noguchi H, Toyoda A, Ogura Y, Okuno M et al. Efficient de novo assembly of highly heterozygous genomes from whole-genome shotgun short reads. Genome research. 2014;24(8):1384-95. doi:10.1101/gr.170720.113.
13. Simpson JT, Wong K, Jackman SD, Schein JE, Jones SJ, Birol I. ABySS: a parallel assembler for short read sequence data. Genome research. 2009;19(6):1117-23. doi:10.1101/gr.089532.108.
14. Price AL, Jones NC, Pevzner PA. De novo identification of repeat families in large genomes. Bioinformatics. 2005;21 Suppl 1:i351-8. doi:10.1093/bioinformatics/bti1018.
15. Xu Z, Wang H. LTR_FINDER: an efficient tool for the prediction of full-length LTR retrotransposons. Nucleic acids research. 2007;35(Web Server issue):W265-8. doi:10.1093/nar/gkm286.
16. Tarailo-Graovac M, Chen N. Using RepeatMasker to identify repetitive elements in genomic sequences. Current protocols in bioinformatics. 2009;Chapter 4:Unit 4 10. doi:10.1002/0471250953.bi0410s25.
17. Bao W, Kojima KK, Kohany O. Repbase Update, a database of repetitive elements in eukaryotic genomes. Mobile DNA. 2015;6:11. doi:10.1186/s13100-015-0041-9.
18. Jurka J, Kapitonov VV, Pavlicek A, Klonowski P, Kohany O, Walichiewicz J. Repbase Update, a database of eukaryotic repetitive elements. Cytogenetic and genome research. 2005;110(1-4):462-7. doi:10.1159/000084979.
19. Stanke M, Keller O, Gunduz I, Hayes A, Waack S, Morgenstern B. AUGUSTUS: ab initio prediction of alternative transcripts. Nucleic acids

research. 2006;34(Web Server issue):W435-9. doi:10.1093/nar/gkl200.

20. Keller O, Kollmar M, Stanke M, Waack S. A novel hybrid gene prediction method employing protein multiple sequence alignments. Bioinformatics. 2011;27(6):757-63. doi:10.1093/bioinformatics/btr010.

21. Majoros WH, Pertea M, Salzberg SL. TigrScan and GlimmerHMM: two open source ab initio eukaryotic gene-finders. Bioinformatics. 2004;20(16):2878-9. doi:10.1093/bioinformatics/bth315.

22. Kent WJ. BLAT--the BLAST-like alignment tool. Genome research. 2002;12(4):656-64. doi:10.1101/gr.229202. Article published online before March 2002.

23. Birney E, Clamp M, Durbin R. GeneWise and Genomewise. Genome research. 2004;14(5):988-95. doi:10.1101/gr.1865504.

24. Elsik CG, Mackey AJ, Reese JT, Milshina NV, Roos DS, Weinstock GM. Creating a honey bee consensus gene set. Genome biology. 2007;8(1):R13. doi:10.1186/gb-2007-8-1-r13.

25. Trapnell C, Pachter L, Salzberg SL. TopHat: discovering splice junctions with RNA-Seq. Bioinformatics. 2009;25(9):1105-11. doi:10.1093/bioinformatics/btp120.

26. Trapnell C, Roberts A, Goff L, Pertea G, Kim D, Kelley DR et al. Differential gene and transcript expression analysis of RNA-seq experiments with TopHat and Cufflinks. Nature protocols. 2012;7(3):562-78. doi:10.1038/nprot.2012.016.

27. Bairoch A, Apweiler R. The SWISS-PROT protein sequence database and its supplement TrEMBL in 2000. Nucleic acids research. 2000;28(1):45-8.

28. Kanehisa M, Goto S, Sato Y, Kawashima M, Furumichi M, Tanabe M. Data, information, knowledge and principle: back to metabolism in KEGG. Nucleic acids research. 2014;42(Database issue):D199-205. doi:10.1093/nar/gkt1076.

29. Kanehisa M, Goto S. KEGG: kyoto encyclopedia of genes and genomes. Nucleic acids research. 2000;28(1):27-30.

30. Jones P, Binns D, Chang HY, Fraser M, Li W, McAnulla C et al. InterProScan 5: genome-scale protein function classification. Bioinformatics. 2014;30(9):1236-40. doi:10.1093/bioinformatics/btu031.

31. Zdobnov EM, Apweiler R. InterProScan--an integration platform for the

signature-recognition methods in InterPro. Bioinformatics. 2001;17(9):847-8.

32. Simao FA, Waterhouse RM, Ioannidis P, Kriventseva EV, Zdobnov EM. BUSCO: assessing genome assembly and annotation completeness with single-copy orthologs. Bioinformatics. 2015;31(19):3210-2. doi:10.1093/bioinformatics/btv351.

33. Fu, Y; Li, L; Hao, S; Guan, R; Fan, G; Shi, C; Wan, H; Chen, W; Zhang, H; Liu, G; Wang, J; Ma, L; You, J; Ni, X; Yue, Z; Xu, X; Sun, X; Liu, X; Lee, S, M (2017): Supporting data for "Draft genome sequence of the Tibetan medicinal herb, Rhodiola crenulata" GigaScience Database. http://dx.doi.org/10.5524/100301

Click here to access/download
Supplementary Material
Additional file1_Supplementary Tables and Figures.DOCX

Click here to access/download
Supplementary Material
Additional file 2_Protocols.io.xlsx

Click here to access/download
Supplementary Material
Additional file3_In-house Perl Script.pdf

Personal Cover Click here to download Personal Cover Cover Letter.docx

Dear Editor,

We are thankful to you and referees for the constructive suggestions and helpful comments. We have implemented all these suggestions and revised the manuscript. Please see the response letter for details.

We used the Illumina data for genome assembly and we used the BGISEQ-500 data for RNA sequencing which aided the gene annotation. Since it's the first time BGISEQ-500 data was used for de novo genome studies (although not for assembly), we have added details on the methods which can serve as reference for future studies. We also included the reference describing the data of BGISEQ-500. In addition, we have removed statements that suggest a proven medical effect of *R. crenulata* in our revised manuscript.

With all these revisions, we hope that the revised manuscript would be suitable for publication in *GigaScience*.

Sincerely yours,

Xin Liu, PhD
liuxin@genomics.cn
BGI-Shenzhen, Shenzhen, 518083, China

We are thankful to the editor and referees for the constructive suggestions and helpful comments. We have implemented all these suggestions and revised the manuscript. With all these revisions, we hope that the revised manuscript would be suitable for publication in *GigaScience*.

Reviewer #1

This is a well-done article on the genome of an interesting species. This will be a valuable resource for researchers studying that species and related species, but also is quite useful as a comparison for many broader studies, since there are no close relatives with sequenced genomes. Regarding the genome size estimates, it would be useful to compare this estimate to estimates for closely related species, and to give more details on the analysis performed. Many estimates for genome size for closely related genera, at least, are available at: http://data.kew.org/cvalues/, for instance.

Response

Thanks for the positive comments. For the genome size estimation, we are thankful to the reviewer for this constructive suggestion. We retrieved all the estimated genome sizes of the species from family *Crassulaceae* in the C-values database mentioned by the reviewer. We found the genome size varies enormously among species from 142 Mb to 8.9 Gb. For *R. crenulata,* our estimated genome size in this study based on kmer analysis is close to the median, 636 Mb.

Also, we have added this information in the revised manuscript and the description of kmer analysis methods in additional file 1 and additional file 3.

Minor comments: In the abstract, 'would be useful' should be 'will be useful'.

Response

Thanks very much. We have corrected it in our revised manuscript.

Reviewer #2

The authors report the generation of high coverage Illumina HiSeq short read sequence data and draft genome assembly for one of the well-known Tibetan medicinal herb, Rhodiola crenulata, with good reasons for drafting genome assembly, including understanding pharmacological mechanisms and resolving issues of adulteration in the market.

To improve the quality of assembly genome, the authors ran many prevalent de novo assemblers with various parameters for comparison and found the most suitable tools from these assemblers.

For the objective of this manuscript, the data sequencing, assembling and analysis are most well organized and documented. As a data note, this manuscript didn't describe any biological questions that were addressed using this genome assembly or any result from comparative analysis. The datasets from this manuscript could provide valuable source for further comparative analysis and answering some biological questions.

Response

We would like to thank this reviewer for the positive comments.

In "Sample collection and Sequencing" section, authors should explain why used multiple sequencing platforms including Illumina HiSeq 2000/4000 platform, and BGISEQ-500 platform.

Response

Generally, different sequencing platforms were used in this project considering about the convenience and effectiveness of the data generation. For the genome assembly, all the sequencing data were generated from Illumina platforms for consistency of data. Considering about data throughput, we used Hiseq2000 to sequence short insert size libraries for more data generation, and Hiseq4000 to sequence the mate pair libraries. In the meantime, we used BGISEQ-500 for RNA sequencing since it’s available and more cost effective. Overall, applying the Illumina data for genome assembly (Hiseq2000 for contig assembly and Hiseq4000 for scaffolding) and BGISEQ-500 data for RNA sequencing, guaranteed the consistency of data, and improved the efficiency

of the study.

Also, we have added the information into the “Sample collection and sequencing” section of our revised manuscript.

Line 31 in "Sample collection and Sequencing" section: These parameters of SOAPfilter are not necessary to be showed here. They were already written in the supplementary spreadsheet (Additional file 2).

Response

Thanks for this suggestion and we have removed it in our revised manuscript.

Line 54-56 in "Sample collection and Sequencing" section: These parameters of SOAPnuke should be moved to supplementary spreadsheet (Additional file 2).

Response

We thank the reviewer for this suggestion, and we have corrected it in our revised manuscript and the additional file 2.
