# Supplementary material for: Draft genome sequence of the Tibetan medicinal herb Rhodiola crenulata
Source: Gigascience. 2017 May 5;6(6):1–5. doi: 10.1093/gigascience/gix033 (PMC5530320; doi:10.1093/gigascience/gix033)
Supplement: Additional File 1: — Supplementary Tables and Figures.docx [file gix033_Additional_file_1_Supplementary_Tables_and_Figures.DOCX]

**Supplementary Tables and Figures**

**Table S1**. Statistics of DNA sequencing data generated by Illumina HiSeq 2000/4000.

| Pair-end Libraries | Inset size | Read length(bp) | Raw data | | High-quality data | |
| --- | --- | --- | --- | --- | --- | --- |
|  |  |  | Total bases | Sequencing depth | Total bases | Sequencing depth |
|  |  |  | (Gb) | (X) | (Gb) | (X) |
| Hiseq 2000 reads | 500 bp | 100_100 | 28.12 | 66.01 | 25.25 | 59.27 |
|  | 800 bp | 100_100 | 26.21 | 61.53 | 23.01 | 54.01 |
| Hiseq 4000 reads | 250 bp | 100_100 | 39.97 | 93.83 | 34.44 | 80.85 |
|  | 5 kb | 100_100 | 25.37 | 59.55 | 18.13 | 42.56 |
|  | 10 kb | 75_75 | 22.1 | 51.88 | 7.36 | 17.28 |
|  | 20 kb | 75_75 | 20.31 | 47.68 | 15.28 | 35.87 |
| Total | ---- | ---- | 162.08 | 380.47 | 123.47 | 289.84 |

**Table S2**. Statistics of RNA sequencing data generated by BGISEQ-500.

| Tissue | Read length(bp) | Raw data | | High-quality data | |
| --- | --- | --- | --- | --- | --- |
|  |  | Total bases | Q20 | Total bases | Q20 |
|  |  | (Gb) |  | (Gb) |  |
| Root | 50 | 4.21 | 92.89% | 4.11 | 93.13% |
| Stem | 50 | 4.10 | 93.05% | 4.01 | 93.31% |
| Flower | 50 | 5.23 | 92.48% | 5.11 | 92.74% |

**Table S3**. Statistics information of 17-mer analysis.

| **K** | **K-mer Number** | **Kmer_Depth** | **Genome Size (bp)** | **Repetitive ratio** | **Heterozygous ratio** |
| --- | --- | --- | --- | --- | --- |
| 17 | 27,736,091,790 | 66 | 420,243,815 | 0.66147454 | 0.01122034 |

* The kmer analysis for genome size estimation[[1-3](#_ENREF_1)]. Briefly, 17-mer (17 bp sub-sequences) from 34.4 Gb genome sequencing data were first extracted and the frequencies of each 17mer was calculated. Considering that majority of the 17mers should be unique in the genome except for highly repetitive regions, the frequencies of these unique 17mers would reflect the sequencing depth, and then we got the genome size with the formula: Genome size = Kmer_num/Kmer_depth. As for the repetitive and heterozygous ratio, in-house Perl script (Additional file 3) was used.

**Table S4.** Statistics information of estimated genome sizes in family *Crassulaceae* according to the Cvalues database (See <http://data.kew.org/cvalues/>).

| Plant group | **Genus** | **Species** | 1C (Mbp) | **Original Reference** | **Paper** |
| --- | --- | --- | --- | --- | --- |
| Angiosperm | Sedum | album | 142 | Hart, 1991 (Ref. 398) | 2005a |
| Angiosperm | Sedum | obtusifolium | 205 | Hart, 1991 (Ref. 398) | 2005a |
| Angiosperm | Sedum | stellatum | 289 | Hart et al., 1993 (Ref. 399) | 2005a |
| Angiosperm | Sedum | stoloniferum | 308 | Hart et al., 1993 (Ref. 399) | 2005a |
| Angiosperm | Sedum | ochroleucum | 445 | Hart, 1991 (Ref. 398) | 2005a |
| Angiosperm | Sedum | forsterianum | 450 | Hart, 1991 (Ref. 398) | 2005a |
| Angiosperm | Greenovia | dodrendalis | 455 | Suda et al., 2005 (Ref. 552) | 2011 |
| Angiosperm | Greenovia | aizoon | 479 | Suda et al., 2005 (Ref. 552) | 2011 |
| Angiosperm | Aeonium | ciliatum | 499 | Suda et al., 2005 (Ref. 552) | 2011 |
| Angiosperm | Monanthes | muralis | 499 | Suda et al., 2005 (Ref. 552) | 2011 |
| Angiosperm | Aeonium | smithii | 504 | Suda et al., 2005 (Ref. 552) | 2011 |
| Angiosperm | Sedum | montanum | 513 | Hart, 1991 (Ref. 398) | 2005a |
| Angiosperm | Aeonium | urbicum | 518 | Suda et al., 2005 (Ref. 552) | 2011 |
| Angiosperm | Aeonium | canariense | 528 | Suda et al., 2005 (Ref. 552) | 2011 |
| Angiosperm | Aeonium | spathulatum | 538 | Suda et al., 2005 (Ref. 552) | 2011 |
| Angiosperm | Aeonium | palmense | 548 | Suda et al., 2005 (Ref. 552) | 2011 |
| Angiosperm | Aeonium | tabulaeforme | 548 | Suda et al., 2005 (Ref. 552) | 2011 |
| Angiosperm | Sedum | sediforme | 567 | Hart, 1991 (Ref. 398) | 2005a |
| Angiosperm | Aeonium | holochrysum | 597 | Suda et al., 2005 (Ref. 552) | 2011 |
| Angiosperm | Aeonium | goochiae | 616 | Suda et al., 2005 (Ref. 552) | 2011 |
| Angiosperm | Monanthes | polyphylla | 636 | Suda et al., 2005 (Ref. 552) | 2011 |
| Angiosperm | Sedum | burrito | **636** | Loureiro et al., 2006 (Ref. 587) | 2011 |
| Angiosperm | Monanthes | laxiflora | 655 | Suda et al., 2005 (Ref. 552) | 2011 |
| Angiosperm | Aeonium | sedifolium | 660 | Suda et al., 2005 (Ref. 552) | 2011 |
| Angiosperm | Aichryson | laxum | 660 | Suda et al., 2005 (Ref. 552) | 2011 |
| Angiosperm | Aeonium | lindleyi | 665 | Suda et al., 2005 (Ref. 552) | 2011 |
| Angiosperm | Aichryson | parlatorei | 670 | Suda et al., 2005 (Ref. 552) | 2011 |
| Angiosperm | Monanthes | brachycaulos | 719 | Suda et al., 2005 (Ref. 552) | 2011 |
| Angiosperm | Aeonium | haworthii | 758 | Hanson et al., 2001 (Ref. 378) | 2005a |
| Angiosperm | Sedum | obtusifolium | 826 | Hart et al., 1993 (Ref. 399) | 2005a |
| Angiosperm | Monanthes | anagensis | 880 | Suda et al., 2005 (Ref. 552) | 2011 |
| Angiosperm | Sedum | rupestre | 1,012 | Hart, 1991 (Ref. 398) | 2005a |
| Angiosperm | Sedum | acre | 1,223 | Nagl et al., 1983 (Ref. 457) | 2005a |
| Angiosperm | Sedum | spurium | 1,731 | Hart et al., 1993 (Ref. 399) | 2005a |
| Angiosperm | Aeonium | simsii | 2,117 | Zonneveld et al., 2005 (Ref. 466) | 2005b |
| Angiosperm | Sedum | hispanicum | 2,641 | Kubesová et al., 2010 (Ref. 630) | 2012 |
| Angiosperm | Sedum | spurium | 2,758 | Hart et al., 1993 (Ref. 399) | 2005a |
| Angiosperm | Graptopetalum | macdougallii | 3,276 | Zonneveld et al., 2005 (Ref. 466) | 2005b |
| Angiosperm | Graptopetalum | bellum | 4,108 | Zonneveld et al., 2005 (Ref. 466) | 2005b |
| Angiosperm | Aeonium | nobile | 4,157 | Zonneveld et al., 2005 (Ref. 466) | 2005b |
| Angiosperm | Sedum | suaveolens | 8,900 | Zonneveld et al., 2005 (Ref. 466) | 2005b |

**Table S5.** Statistics of the assembly with different assemblers.

| **Assembler** | **Assembly size (bp)** | **Length of Ns**  **(bp)** | **Contig N50**  **(bp)** | **Scaffold N50**  **(bp)** | **K-mer**  **(bp)** | | **Gapcloser** | |
| --- | --- | --- | --- | --- | --- | --- | --- | --- |
| ABySS | 733,589,498 | 134,616,235 | 2,049 | 6,199 | | 75 | | Yes |
| ABySS | 561,884,304 | 43,720,836 | 353 | 1,439 | | 55 | | No |
| SOAP*denovo*2 | 729,083,825 | 251,758,467 | 5,322 | 87,781 | | 35 | | Yes |
| SOAP*denovo2* | 440,349,280 | 51,697,986 | --- | 1,898 | | 56 | | No |
| SOAP*denovo2* | 141,634,012 | 3,867,383 | --- | 394 | | 71 | | No |
| Platanus | 353,437,818 | 87,011,092 | 2,886 | 148,240 | | 35 | | No |
| Platanus | 357,217,744 | --- | --- | 127,404 | | 55 | | No |
| Platanus | 362,529,844 | --- | --- | 134,265 | | 65 | | No |
| Platanus | 376,172,948 | --- | --- | 137,210 | | 73 | | No |
| Platanus | 345,125,290 | 65,911,704 | 6,301 | 145,079 | | 35 | | No |
| **Platanus** | **344,513,827** | **25,706,707** | **25,360** | **144,749** | | **35** | | **Yes** |

**Table S6.** The statistics of repeats predicted in *de novo* methods.

| **Type** | **Length (bp)** | **Percentage of genome (%)** |
| --- | --- | --- |
| DNA | 25,204,257 | 7.32 |
| LINE | 9,027,376 | 2.62 |
| SINE | 361,579 | 0.10 |
| LTR | 133,619,487 | 38.78 |
| Satellite | 215,398 | 0.06 |
| Simple_repeat | 3,303,778 | 0.96 |
| Unknown | 11,522,413 | 3.34 |
| Total | 175,048,756 | 50.81 |

**Table S7.** The statistics of transposable elements predicted in a combination of the *de novo* and homolog-based methods.

| **Type** | **RepeatModeler** | | **RepeatProteinMask** | | ***De novo*** | | **Combined TEs** | |
| --- | --- | --- | --- | --- | --- | --- | --- | --- |
|  | **Length (bp)** | **% in genome** | **Length (bp)** | **% in genome** | **Length (bp)** | **% in genome** | **Length (bp)** | **% in genome** |
| DNA | 5,347,888 | 1.55 | 7,559,547 | 2.19 | 25,204,257 | 7.32 | 34,392,427 | 9.98 |
| LINE | 1,497,308 | 0.43 | 6,495,442 | 1.89 | 9,027,376 | 2.62 | 15,870,025 | 4.61 |
| SINE | 12,249 | 0.0036 | 0 | 0 | 361,579 | 0.10 | 372,702 | 0.11 |
| LTR | 27,604,630 | 8.01 | 41,754,549 | 12.12 | 133,619,487 | 38.78 | 174,555,674 | 50.67 |
| Other | 507 | 0.0001 | 0 | 0 | 0 | 0 | 507 | 0.0001 |
| Unknown | 0 | 0 | 11,265 | 0.003 | 11,522,413 | 3.34 | 11,533,678 | 3.35 |
| Total | 34,188,933 | 9.92 | 55,803,522 | 16.20 | 171,529,580 | 49.79 | 226,594,616 | 65.77 |

**Table S8.** The statistics of predicted gene models.

| Gene set | | Number | Average transcript length (bp) | Average CDS length (bp) | Average exon per gene | Average exon length (bp) | Average intron length (bp) |
| --- | --- | --- | --- | --- | --- | --- | --- |
| *De novo* | Augustus | 31,005 | 2,267 | 1168 | 5.36 | 218 | 252 |
|  | GlimmerHMM | 34,586 | 1,865 | 978 | 4.18 | 234 | 279 |
|  | *A. thaliana* | 36,495 | 2,419 | 1197 | 6.11 | 196 | 239 |
| Homolog | *F. vesca* | 27,034 | 2,387 | 1234 | 5.78 | 214 | 241 |
|  | *P. mume* | 28,767 | 2,335 | 1207 | 5.66 | 213 | 242 |
|  | *P. persica* | 25,976 | 2,198 | 1146 | 5.31 | 216 | 244 |
| GLEAN | | 27,107 | 2,174 | 1169 | 4.95 | 236 | 254 |
| Final | | 31,517 | 2,202 | 1044 | 4.37 | 239 | 253 |

**Table S9**. The statistics of functional annotation

|  | **Database** | **Number** | **Percentage (%)** |
| --- | --- | --- | --- |
| Total |  | 31,517 | 100 |
|  | InterPro | 18,687 | 59.29 |
|  | GO | 13,922 | 44.17 |
|  | KEGG | 19,124 | 60.68 |
|  | Swissprot | 19,357 | 61.42 |
|  | TrEMBL | 24,864 | 78.89 |
| Annotated |  | 25,128 | 79.73 |

**Table S10**. Statistics of the mapping ratio of RNA sequencing reads.

| **Tissue** | | **Number of input reads** | | **Number of mapped reads** | **Percentage(%)** |
| --- | --- | --- | --- | --- | --- |
| Root | | 82,308,621 | 66,790,422 | 81.15% |  |
| Stem | | 81,704,634 | 66,671,495 | 81.60% |  |
| Leaf | | 104,126,971 | 85,351,619 | 81.97% |  |

**Fig. S1.**

The 17-mer depth distribution of *Rhodiola crenulata*

1. Luo R, Liu B, Xie Y, Li Z, Huang W, Yuan J et al. SOAPdenovo2: an empirically improved memory-efficient short-read de novo assembler. GigaScience. 2012;1(1):18. doi:10.1186/2047-217X-1-18.

2. Li R, Fan W, Tian G, Zhu H, He L, Cai J et al. The sequence and de novo assembly of the giant panda genome. Nature. 2010;463(7279):311-7. doi:10.1038/nature08696.

3. Varshney RK, Chen W, Li Y, Bharti AK, Saxena RK, Schlueter JA et al. Draft genome sequence of pigeonpea (Cajanus cajan), an orphan legume crop of resource-poor farmers. Nature biotechnology. 2011;30(1):83-9. doi:10.1038/nbt.2022.
