# Supplementary material for: Draft genome sequence of the Tibetan medicinal herb Rhodiola crenulata
Source: Gigascience. 2017 May 5;6(6):1–5. doi: 10.1093/gigascience/gix033 (PMC5530320; doi:10.1093/gigascience/gix033)
Supplement: Additional_file_3_In-house_Perl_Script.pdf [file gix033_Additional_file_3_In-house_Perl_Script.pdf]

```perl
use strict;
use warnings;

die "perl $0 <kmer.xls> <k> <kmer_num> <expect_peak:default is the most high peak>\nplease see the example before you use this script\n" if(@ARGV==0);

# global variables
my $REPEAT = 1.6;
my @kmers;
my $size;
my @peaks;
my $kmer_depth;
my $repeat;
my $hete;
my $read_coverage;
my $error_rate;
my $kmer_error;

my($data,$k,$kmer_num,$manual_peak) = @ARGV;
@peaks = getPeaks($data);
my($c1,$p1,$n1) = &getFirstLine($data);

foreach my $peak(@peaks){
	print "\n************************************************\n";
	$kmer_depth = $peak;
	$size = &sizeGuess();
	$repeat = &repeatGuess($kmer_num,$kmer_depth,$size);
	$hete = &heteGuess();
	&debug();
}

$manual_peak||=$kmer_depth;
if($manual_peak!=$kmer_depth){
	print "\n--------------------manual set--------------------\n";
	$kmer_depth = $manual_peak;
	$size = &getSize1($kmer_num,$kmer_depth);
	$repeat = &repeatGuess($kmer_num,$kmer_depth,$size);
	$hete = &heteGuess();
	&debug();
}
############################# sub routine ###########################
sub debug{
	print "peak:$kmer_depth\n";
	print "genomeSize:$size\n";
```

```perl
	print "repeat:$repeat\n";
	print "hete:$hete\n";
}
sub sizeGuess{
	my $size = &getSize1($kmer_num,$kmer_depth);
	return $size;
}
sub repeatGuess{
	my($kmer_total,$kmer_depth,$size)=@_;
	my $num_unique = 0;
	for(my $i=0;$i<$REPEAT*$kmer_depth;$i++){
		my($count,$percent,$num) = split(/\t/,$kmers[$i]);
		$num_unique += $count*$num;
	}
	my $repeat = ($kmer_total - $num_unique)/$kmer_depth/$size;
	$repeat = $repeat/0.986;
	return $repeat;
}
sub heteGuess1{
	my $inflex = inflexion();
	$inflex = 2 if($inflex>=0.5*$kmer_depth);
	my $max = $REPEAT*$kmer_depth;
	my $kmer_uni = 0;
	for(my $i=$inflex+1;$i<$max;$i++){
		my($count,$percent,$num) = split(/\t/,$kmers[$i]);
		$kmer_uni += $num;
	}
	my($count,$percent,$num_peak) = split(/\t/,$kmers[$kmer_depth]);
	my $kmer_org = $size*(1-$repeat); # unique kmers
	my $possion = getPossion($kmer_depth,$kmer_depth);
	my $result = 1-($num_peak/$kmer_org/$possion)**(1/$k);
	return $result;
}

sub heteGuess{
	my $inflex = inflexion();
	$inflex = 2 if($inflex>=0.5*$kmer_depth);
	my $max = $REPEAT*$kmer_depth;
	my $kmer_uni = 0;
	for(my $i=$inflex+1;$i<$max;$i++){
		my($count,$percent,$num) = split(/\t/,$kmers[$i]);
		$kmer_uni += $num;
	}
	my($count,$percent,$num_peak) = split(/\t/,$kmers[$kmer_depth]);
```

```
	my $kmer_org = $size*(1-$repeat); # unique kmers
	my $add_percent = ($kmer_uni-$kmer_org)/$kmer_org;#
	my $result =1-(1-$add_percent)**(1/$k);
	$result = 0 if($result<=0);
	return $result;
}

sub getPeaks{
	my $CUT = 0.0005;
	my($file) = @_;
	open FI,"$file" or die $!;
	my @peaks;
	while(my $line=<FI>){
		chomp $line;
		my($count,$percent,$num) = split(/\t/,$line);
		push(@kmers,"$count\t$percent\t$num");
	}
	close FI;

	for(my $i = 1;$i<$#kmers-1;$i++){
		my($count1,$percent1,$num1) = split(/\t/,$kmers[$i-1]);
		my($count2,$percent2,$num2) = split(/\t/,$kmers[$i]);
		my($count3,$percent3,$num3) = split(/\t/,$kmers[$i+1]);
		push(@peaks,$count2)   if($percent2>$percent1   &&   $percent2>$percent3   &&
$percent2>$CUT);
	}
	return @peaks;
}

sub getFirstLine{
	my($file) = @_;
	open FI,"$file" or die $!;
	my $line = <FI>;
	chomp $line;
	my @array = split(/\t/,$line);
	close FI;
	return @array;
}

sub inflexion{
	my $num_err = 0;
	for(my $i = 1;$i<$#kmers-1;$i++){
		my($count1,$percent1,$num1) = split(/\t/,$kmers[$i-1]);
		my($count2,$percent2,$num2) = split(/\t/,$kmers[$i]);
```

```
		my($count3,$percent3,$num3) = split(/\t/,$kmers[$i+1]);
		return $count2 if($percent2<$percent1 && $percent2<$percent3);
	}
	return 2;
}

sub getSize1{
	my $inflexion = &inflexion();
	my($kmer_num,$kmer_exp) = @_;
	$inflexion = 2 if($inflexion>=0.5*$kmer_exp);
	# get the error kmer number
	my $kmer_e =0;
	for(my $i=0;$i<=$inflexion;$i++){
		my($count,$percent,$num) = split(/\t/,$kmers[$i]);
		$kmer_e += $count*$num;
	}
	$kmer_error = $kmer_e;
	my $kmer_cor = $kmer_num-$kmer_e;
	my $size = int($kmer_cor/$kmer_exp);
	return $size;
}
```
