# Supplementary material for: Draft genome sequence of the Tibetan medicinal herb Rhodiola crenulata
Source: Gigascience. 2017 May 5;6(6):1–5. doi: 10.1093/gigascience/gix033 (PMC5530320; doi:10.1093/gigascience/gix033)
Supplement: Response_to_reviewer_comments_original_submission_.pdf [file gix033_Response_to_reviewer_comments_original_submission_.pdf]

We are thankful to the editor and referees for the constructive suggestions and helpful comments. We used the Illumina data for genome assembly and we used the BGISEQ-500 data for RNA sequencing which aided the gene annotation. Since it's the first time BGISEQ-500 data was used for de novo genome studies (although not for assembly), we have added details on the methods which can serve as reference for future studies. We also included the reference describing the data of BGISEQ-500. With all these revisions, we hope that the revised manuscript would be suitable for publication in GigaScience.

Reviewer #1
This is a well-done article on the genome of an interesting species. This will be a valuable resource for researchers studying that species and related species, but also is quite useful as a comparison for many broader studies, since there are no close relatives with sequenced genomes. Regarding the genome size estimates, it would be useful to compare this estimate to estimates for closely related species, and to give more details on the analysis performed. Many estimates for genome size for closely related genera, at least, are available at: http://data.kew.org/cvalues/, for instance.

Response
Thanks for the positive comments. For the genome size estimation, we are thankful to the reviewer for this constructive suggestion. We retrieved all the estimated genome sizes of the species from family Crassulaceae in the C-values database mentioned by the reviewer. We found the genome size varies enormously among species from 142 Mb to 8.9 Gb. For R. crenulata, our estimated genome size in this study based on kmer analysis is close to the median, 636 Mb.
Also, we have added this information in the revised manuscript and the description of kmer analysis methods in additional file 1 and additional file 3.

Minor comments: In the abstract, 'would be useful' should be 'will be useful'.
Response
Thanks very much. We have corrected it in our revised manuscript.

Reviewer #2
The authors report the generation of high coverage Illumina HiSeq short read sequence data and draft genome assembly for one of the well-known Tibetan medicinal herb, Rhodiola crenulata, with good reasons for drafting genome assembly, including understanding pharmacological mechanisms and resolving issues of adulteration in the market.

To improve the quality of assembly genome, the authors ran many prevalent de novo assemblers with various parameters for comparison and found the most suitable tools from these assemblers.

For the objective of this manuscript, the data sequencing, assembling and analysis are most well organized and documented. As a data note, this manuscript didn't describe any biological questions that were addressed using this genome assembly or any result from comparative analysis. The datasets from this manuscript could provide valuable source for further comparative analysis and answering some biological questions.

Response
We would like to thank this reviewer for the positive comments.

In "Sample collection and Sequencing" section, authors should explain why used multiple sequencing platforms including Illumina HiSeq 2000/4000 platform, and BGISEQ-500 platform.
Response
Generally, different sequencing platforms were used in this project considering about the convenience and effectiveness of the data generation. For the genome assembly, all the sequencing data were generated from Illumina platforms for consistency of data. Considering about data throughput, we used Hiseq2000 to sequence short insert size libraries for more data generation, and Hiseq4000 to sequence the mate pair libraries. In the meantime, we used BGISEQ-500 for RNA sequencing since it's available and more cost effective. Overall, applying the Illumina data for genome assembly (Hiseq2000 for contig assembly and Hiseq4000 for scaffolding) and BGISEQ-500 data for RNA sequencing, guaranteed the consistency of data, and improved the efficiency of the study.
Also, we have added the information into the "Sample collection and sequencing" section of our revised manuscript.

Line 31 in "Sample collection and Sequencing" section: These parameters of SOAPfilter are not necessary to be showed here. They were already written in the supplementary spreadsheet (Additional file 2).
Response
Thanks for this suggestion and we have removed it in our revised manuscript.

Line 54-56 in "Sample collection and Sequencing" section: These parameters of SOAPnuke should be moved to supplementary spreadsheet (Additional file 2).
Response
We thank the reviewer for this suggestion, and we have corrected it in our revised manuscript and the additional file 2.
