# Supplementary material for: Draft genome sequence of the Tibetan medicinal herb Rhodiola crenulata
Source: Gigascience. 2017 May 5;6(6):1–5. doi: 10.1093/gigascience/gix033 (PMC5530320; doi:10.1093/gigascience/gix033)
Supplement: Reviewer_3_Report_(Original_Submission).pdf [file gix033_Reviewer_3_Report_(Original_Submission).pdf]

Reviewer Report

Title: "A dataset of images and morphological profiles of 30,000 small-molecule treatments using the Cell Painting assay"

Version: Original Submission **Date:** 3/30/2016

Reviewer name: Click here to enter text.

Reviewer Comments to Author:

The manuscript by Bray et al provides a complete data set derived from the Carpenter lab's Cell Painting assay analysis of a 30,000 compound small molecule library, including primary image data and associated metadata (compound ID information and image curation statistics.) The data set also includes the analysis pipelines for CellProfiler, the open source high-content image analysis package created by the Carpenter lab used in the original analysis of these data presented in an earlier manuscript. Additionally, the data set includes intermediate processing steps, particularly the shading correction images that are produced as the output of an initial processing pipeline and the final data output for each plate.

The manuscript and associated supplemental text provide a clear and sufficiently-detailed roadmap to the data and metadata together with an overview of the Cell Painting method. Additional links to the Carpenter lab Git repository and the Cell Painting Wiki provide critical, up-to-date information not found in the manuscript.

These data are extremely useful in several respects. First, as the authors suggest, the availability of these primary data allow for subsequent analysis and mining of the data, which may yield additional insights, particularly as there are alternate methods for aggregating the cell-based data arrays. Second, these data provide an opportunity for new users of the Cell Painting method to test their analysis pipeline against results from a previously-analyzed image set.

The data set provides sufficient information for both of these objectives and is a success at this level. A few minor improvements, listed below, might enhance the accessibility of the data set overall.

1. The image data presented in the Cell Image Library (reference 10) does not include all of the plate images described in the GigaDB submission. My efforts to resolve this, using the plate list in the image_curation_statistics.csv file found 60 plate IDs that did not have a corresponding image set (the list of plate IDs that lacked image data is attached *).

2. The pipelines in the GigaDB submission were designed for an earlier version of CellProfiler. The versions on the Cell Painting Wiki were up to date and should be included in the final submission to GigaDB.

3. The pipelines did not work correctly with the image data. In particular, the regular expression parser for metadata in the file directory path was designed for a different file format. This should be updated to work with the image sets deposited on cellimagelibrary.org (reference 10).

Aside from these minor comments, the manuscript and associated data are thorough, extremely useful, and well suited for publication in this forum.

*Plate IDs lacking associated image data

24277

24280

24293

24294

24295

24296

24300

24301

24302

24303

24305

24306

24308

24309

24310

24311

24312

24313

24320

24321

24357

24508

24509

24514

24515

24516

24517

24518

24523

24525

24591

24635

24636

24637

24638

24639

24640

24643

24644

24645

24646

24647

24648

24651

24652

24654

24655

24656

24657

24661

24663

24666

24667

24683

24684

24685

24687

24688

26795

26796

Level of Interest

Please indicate how interesting you found the manuscript: An article of importance in its field

Quality of Written English

Please indicate the quality of language in the manuscript: Acceptable

Declaration of Competing Interests

Please complete a declaration of competing interests, considering the following questions:

- Have you in the past five years received reimbursements, fees, funding, or salary from an organisation that may in any way gain or lose financially from the publication of this manuscript, either now or in the future?
- Do you hold any stocks or shares in an organisation that may in any way gain or lose financially from the publication of this manuscript, either now or in the future?
- Do you hold or are you currently applying for any patents relating to the content of the manuscript?
- Have you received reimbursements, fees, funding, or salary from an organization that holds or has applied for patents relating to the content of the manuscript?
- Do you have any other financial competing interests?
- Do you have any non-financial competing interests in relation to this paper?

If you can answer no to all of the above, write 'I declare that I have no competing interests' below. If your reply is yes to any, please give details below.

I declare that I have no competing interests.

I agree to the open peer review policy of the journal. I understand that my name will be included on my report to the authors and, if the manuscript is accepted for publication, my named report including any attachments I upload will be posted on the website along with the authors' responses. I agree for my report to be made available under an Open Access Creative Commons CC-BY license (http://creativecommons.org/licenses/by/4.0/). I understand that any comments which I do not wish to be included in my named report can be included as confidential comments to the editors, which will not be published.

I agree to the open peer review policy of the journal
