# Supplementary material for: Draft genome sequence of the Tibetan medicinal herb Rhodiola crenulata
Source: Gigascience. 2017 May 5;6(6):1–5. doi: 10.1093/gigascience/gix033 (PMC5530320; doi:10.1093/gigascience/gix033)
Supplement: Review_1_Report_(Original_Submission).pdf [file gix033_Review_1_Report_(Original_Submission).pdf]

Reviewer Report

Title: "Draft genome sequence of the Tibetan medicinal herb, Rhodiola crenulata"

Version: Original Submission **Date:** 3/3/2017

Reviewer name: Nolan Kane

Reviewer Comments to Author:

This is a well-done article on the genome of an interesting species. This will be a valuable resource for researchers studying that species and related species, but also is quite useful as a comparison for many broader studies, since there are no close relatives with sequenced genomes. Regarding the genome size estimates, it would be useful to compare this estimate to estimates for closely related species, and to give more details on the analysis performed. Many estimates for genome size for closely related genera, at least, are available at:http://data.kew.org/cvalues/for instance.Minor comments:in the abstract, 'would be useful' should be 'will be useful'

Level of Interest

Please indicate how interesting you found the manuscript: An article whose findings are important to those with closely related research interests

Quality of Written English

Please indicate the quality of language in the manuscript: Needs some language corrections before being published

Declaration of Competing Interests

Please complete a declaration of competing interests, considering the following questions:

I declare that I have no competing interests

I agree to the open peer review policy of the journal. I understand that my name will be included on my report to the authors and, if the manuscript is accepted for publication, my named report including any attachments I upload will be posted on the website along with the authors' responses. I agree for my report to be made available under an Open Access Creative Commons CC-BY license (http://creativecommons.org/licenses/by/4.0/). I understand that any comments which I do not wish to be included in my named report can be included as confidential comments to the editors, which will not be published.

I agree to the open peer review policy of the journal
