# Supplementary material for: Draft genome sequence of the Tibetan medicinal herb Rhodiola crenulata
Source: Gigascience. 2017 May 5;6(6):1–5. doi: 10.1093/gigascience/gix033 (PMC5530320; doi:10.1093/gigascience/gix033)
Supplement: Review_2_Report_(Original_Submission).pdf [file gix033_Review_2_Report_(Original_Submission).pdf]

Reviewer Report

Title: "Draft genome sequence of the Tibetan medicinal herb, Rhodiola crenulata"Original Submission

Version: Original Submission **Date:** 3/26/2017

Reviewer name: Kaitao Lai, Ph.D.

Reviewer Comments to Author:

The authors report the generation of high coverage Illumina HiSeq short read sequence data and draft genome assembly for one of the well-known Tibetan medicinal herb, Rhodiola crenulata, with good reasons for drafting genome assembly, including understanding pharmacological mechanisms and resolving issues of adulteration in the market.To improve the quality of assembly genome, the authors ran many prevalent de novo assemblers with various parameters for comparison and found the most suitable tools from these assemblers.For the objective of this manuscript, the data sequencing, assembling and analysis are most well organized and documented. As a data note, this manuscript didn't describe any biological questions that were addressed using this genome assembly or any result from comparative analysis. The datasets from this manuscript could provide valuable source for further comparative analysis and answering some biological questions.Minor revisions:In "Sample collection and Sequencing" section, authors should explain why used multiple sequencing platforms including Illumina HiSeq 2000/4000 platform, and BGISEQ-500 platform.Line 31 in "Sample collection and Sequencing" section: These parameters of SOAPfilter are not necessary to be showed here. They were already written in the supplementary spreadsheet (Additional file 2).Line 54-56 in "Sample collection and Sequencing" section: These parameters of SOAPnuke should be moved to supplementary spreadsheet (Additional file 2).

Level of Interest

Please indicate how interesting you found the manuscript: An article of importance in its field

Quality of Written English

Please indicate the quality of language in the manuscript: Acceptable

Declaration of Competing Interests

I declare that I have no competing interests.

I agree to the open peer review policy of the journal. I understand that my name will be included on my report to the authors and, if the manuscript is accepted for publication, my named report including any attachments I upload will be posted on the website along with the authors' responses. I agree for my report to be made available under an Open Access Creative Commons CC-BY license

(http://creativecommons.org/licenses/by/4.0/). I understand that any comments which I do not wish to be included in my named report can be included as confidential comments to the editors, which will not be published.

I agree to the open peer review policy of the journal
